# CXCL12 ameliorates neutrophilia and disease severity in SARS-CoV-2 infection

**DOI:** 10.1172/JCI188222

**Published:** 2025-01-07

**Authors:** Jian Zheng, Hima Dhakal, Enya Qing, Rejeena Shrestha, Anne E. Geller, Samantha M. Morrissey, Divyasha Saxena, Xiaoling Hu, Hong Li, Haiyan Li, Kevin Wilhelmsen, Linder H. Wendt, Klaus Klumpp, Patrick S. Hume, William J. Janssen, Rachel Brody, Kenneth E. Palmer, Silvia M. Uriarte, Patrick Ten Eyck, David K. Meyerholz, Michael L. Merchant, Kenneth McLeish, Tom Gallagher, Jiapeng Huang, Jun Yan, Stanley Perlman

**Affiliations:** 1 Department of Microbiology and Immunology and; 2 Center for Predictive Medicine, University of Louisville, Louisville, Kentucky, USA.; 3 Department of Microbiology and Immunology, University of Iowa, Iowa City, Iowa, USA.; 4 Department of Microbiology and Immunology, Loyola University Chicago, Maywood, Illinois, USA.; 5 Division of Immunotherapy, the Hiram C. Polk, Jr., MD, Department of Surgery, Immuno-Oncology Program, Brown Cancer Center and; 6 Functional Immunomics Core, Brown Cancer Center, University of Louisville, Louisville, Kentucky, USA.; 7 BIOAGE Labs, Richmond, California, USA.; 8 Institute for Clinical and Translational Science, University of Iowa, Iowa City, Iowa, USA.; 9 Department of Medicine, Division of Pulmonary and Critical Care, National Jewish Health, Denver, Colorado, USA.; 10 Department of Medicine, Division of Pulmonary Sciences and Critical Care Medicine, University of Colorado, Aurora, Colorado, USA.; 11 Department of Pathology, Molecular and Cell-Based Medicine, Icahn School of Medicine at Mount Sinai, New York, New York, USA.; 12 Department of Pharmacology and Toxicology and; 13 Department of Oral Immunology and Infectious Diseases, University of Louisville, Louisville, Kentucky, USA.; 14 Department of Pathology, University of Iowa, Iowa City, Iowa, USA.; 15 Department of Medicine, Division of Nephrology and Hypertension and; 16 Department of Anesthesiology and Perioperative Medicine, University of Louisville, Louisville, Kentucky, USA.

**Keywords:** COVID-19, Infectious disease, Chemokines, Endothelial cells, Neutrophils

## Abstract

Neutrophils, particularly low-density neutrophils (LDNs), are believed to contribute to acute COVID-19 severity. Here, we showed that neutrophilia can be detected acutely and even months after SARS-CoV-2 infection in patients and mice, while neutrophil depletion reduced disease severity in mice. A key factor in neutrophilia and severe disease in infected mice was traced to the chemokine CXCL12 secreted by bone marrow cells and unexpectedly, endothelial cells. CXCL12 levels were negatively correlated with LDN numbers in longitudinal analyses of patient blood samples. CXCL12 blockade in SARS-CoV-2–infected mice increased blood/lung neutrophil numbers, thereby accelerating disease progression without changing lung virus titers. The exaggerated mortality caused by CXCL12 blockade could be reversed by neutrophil depletion. In addition, blocking interactions between SARS-CoV-2 and angiotensin-converting enzyme 2 (ACE2) reduced CXCL12 levels, suggesting a signal transduction from virus-mediated ACE2 ligation to increased CXCL12 secretion. Collectively, these results demonstrate a previously unappreciated role of CXCL12 in diminishing neutrophilia, including low-density neutrophilia, and its deleterious effects in SARS-CoV-2 infections. The results also support the involvement of SARS-CoV-2–endothelial cell interactions in viral pathogenesis.

## Abstract 2

CXCL12 secreted by bone marrow and endothelial cells ameliorates SARS-CoV-2 infection by diminishing the accumulation of neutrophils, especially low-density neutrophils, in the blood and lungs.

## Graphical abstract

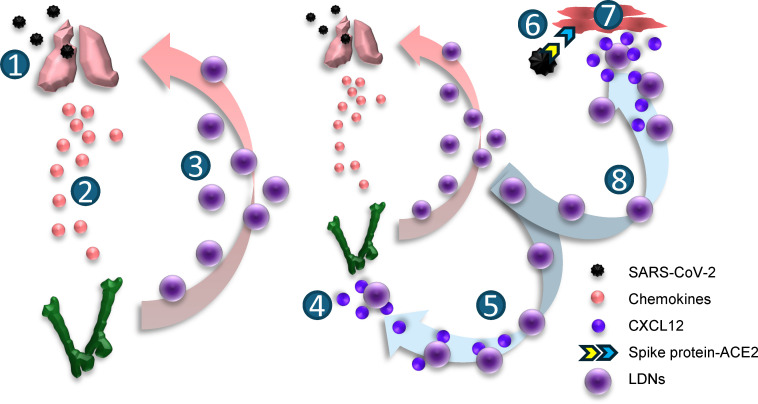

## Introduction

SARS-CoV-2, the etiological agent of COVID-19, causes respiratory disease of varying severity, ranging from asymptomatic infection to death (1). Severe disease, which includes hospitalization, ICU admission, and death, is characterized by a dysfunctional immune response (2, 3), which is correlated with a poor type 1 IFN response in some patients (4). More generally, these patients demonstrate a prolonged innate immune response, with elevated levels of a group of proinflammatory mediators, including IL-6 (5). Notably, severe COVID-19 is often associated with elevated neutrophil counts in the blood (6, 7), and this is often accompanied by lymphopenia (8). Within the increased circulating neutrophil population in SARS-CoV-2–infected patients, a subset of low-density neutrophils (LDNs) (CD11b^+^CD66b^+^CD16^int^) is specifically increased (9). These cells were initially identified in patients with systemic lupus erythematosus using Ficoll-Hypaque gradient centrifugation (10) and their appearance was stress related (11). Unlike mature neutrophils, they have the same density as mononuclear cells after density centrifugation. This LDN subset expresses high levels of proinflammatory cytokines and chemokines upon stimulation, likely contributing to the inflammatory milieu in SARS-CoV-2–infected patients (12–14). Functionally, LDNs from COVID-19 patients showed impaired respiratory burst activity and degranulation, indicative of an immature functional phenotype (15). Previous studies have also identified immature LDNs in circulation and lungs of COVID-19 patients (16–20), indicating that severe COVID-19 is associated with the emergence of less mature LDN populations in the circulation and in the bronchoalveolar lavage fluid (BALF), likely due to emergency myelopoiesis (21). In support of this, other reports showed that aging enhances emergency myelopoiesis (22–24). Together, these results suggest that during stress, such as severe infection, increased numbers of immature neutrophils are expected. Additionally, LDN activation leads to abundant neutrophil extracellular trap (NET) formation, which is associated with COVID-19 progression (25, 26). Of note, LDNs are immunosuppressive in some settings and include granulocytic myeloid-derived suppressor cells (G-MDSCs). G-MDSCs were identified in patients with COVID-19 (18, 27). Neutrophilia persists in some COVID-19 survivors, suggesting a possible relationship with post-acute sequelae of COVID-19 (PASC) (25, 28). Although mechanistic studies are difficult or impossible to perform in patients, the basis and functional relevance of neutrophilia can be addressed in experimentally infected animals, despite differences between human and murine neutrophils (29).

Mice, hamsters, and nonhuman primates are susceptible to SARS-CoV-2 infection (30–33). Mice are not susceptible to infection with ancestral strains of SARS-CoV-2, although they can be infected with many recent variants (34). The incompatibility between mouse ACE2 (mACE2) and the ancestral SARS-CoV-2 spike (S) glycoprotein is rectified by 1 or 2 amino acid changes in the S protein or in mACE2. Therefore, we and others developed mouse-adapted versions of the ancestral strain by mutating the S protein (13, 32, 35). To generate a virulent mouse-adapted SARS-CoV-2, we inserted the N501Y mutation into the SARS-CoV-2 genome using reverse genetics and passaged this mutant virus through mouse lungs (32). After 30 passages, the virus (SARS2-N501Y_MA30_) became highly virulent such that 5000 PFU caused lethal disease in young BALB/c mice. SARS2-N501Y_MA30_ infection resulted in age-dependent pathogenesis in C57BL/6N mice, similar to the age dependence observed in patients.

The present study used a cohort of COVID-19 patients and a mouse model of COVID-19 in which middle-aged (8- to 10-month-old) mice were infected with SARS2-N501Y_MA30_ to examine the role of LDNs in acute COVID-19 and the persistence of neutrophilia after recovery from acute infection. We confirmed the presence of neutrophilia and increased LDNs in COVID-19 patients and infected mice and showed that they also persisted for months after acute infection. We identified a critical role for a single chemokine, CXCL12, in controlling neutrophilia in mice. Additionally, increased plasma CXCL12 levels correlated with decreased numbers of circulating LDNs in a subset of COVID-19 patients who ultimately survived their disease.

## Results

### Neutrophilia with increased LDNs is present in acutely ill and convalescent COVID-19 patients.

**Figure 1:**
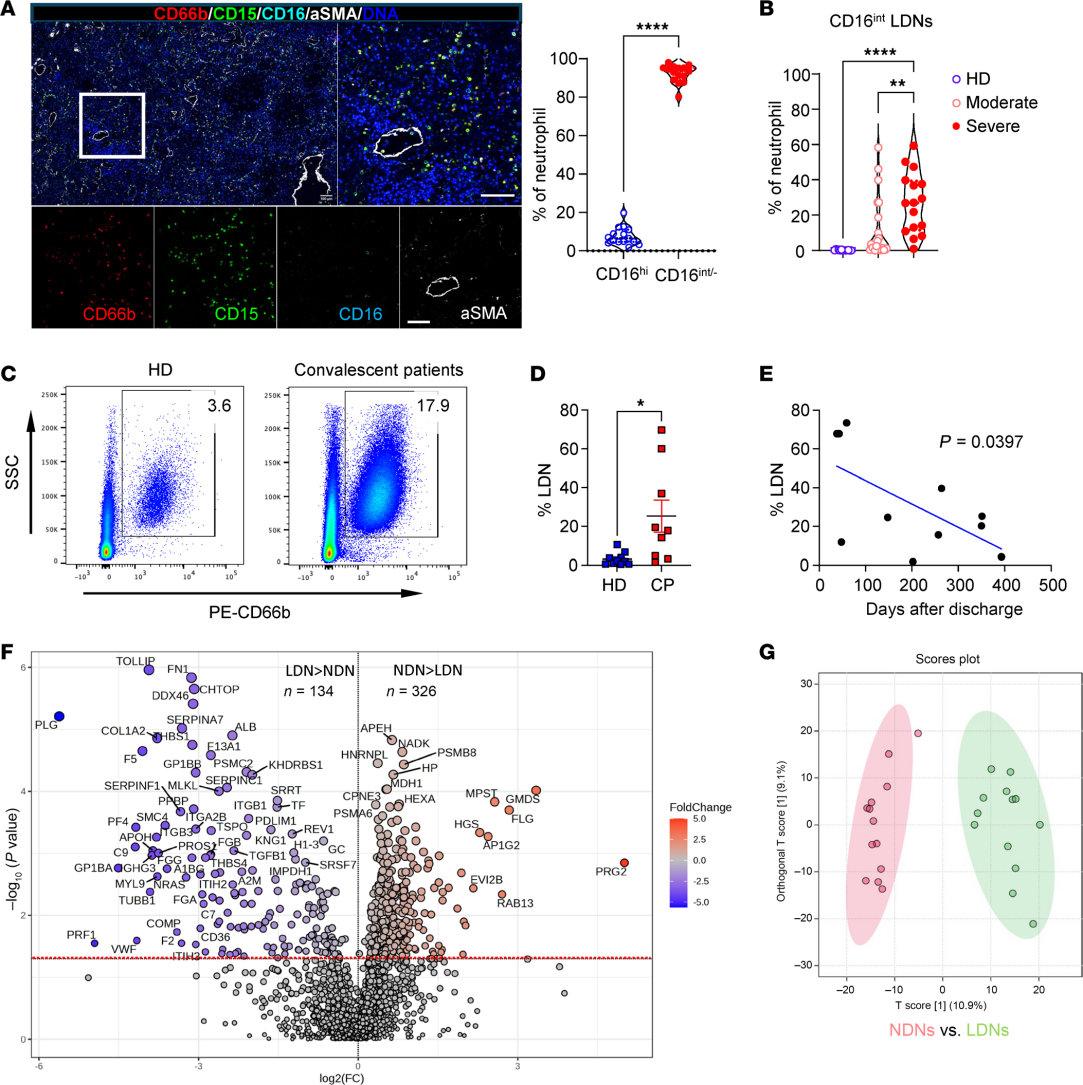
Neutrophilia and accumulated LDNs in COVID-19 patients. (**A**) Massive infiltration of LDNs in the lungs of deceased COVID-19 patients was identified with metal isotope–labeled antibodies (left and right panels). Images are representative of 5 slides from 5 deceased COVID-19 cases. Scale bars: 100 μm. The percentage of CD16^hi^ and CD16^int^ neutrophils in 3 regions of interest (ROIs) of each slide (middle panel) was quantitated by FlowJo after converting imaging files into fcs files. ****P < 0.0001 by Student’s *t* test. (**B**) Peripheral blood LDNs in healthy donors (HD, *n* = 13) and COVID-19 patients with moderate (*n* = 23) or severe disease (*n* = 16). **P < 0.01; ****P < 0.0001 by 1-way ANOVA with Tukey’s multiple comparisons. (**C**–**E**) A cohort of convalescent COVID-19 patients and healthy donors were recruited at times ranging from 1 month to 13 months after hospital discharge. A representative flow plot (**C**) and summary (**D**) of CD66b^+^ LDN frequency in the peripheral blood of convalescent patients (CP, collected at 1–13 months after discharge) and age-matched healthy donors (HD) are shown. *n* = 11. *P < 0.05 by Student’s *t* test. (**E**) Frequency of LDNs was negatively correlated with time from discharge (each point represents the data obtained from an individual patient). (**F** and **G**) A total of 1830 proteins were identified by mass spectrometry of normal-density neutrophils (NDNs) and low-density neutrophils (LDNs) analyzed from each of 13 patients with severe COVID-19. Proteins were quantified from average peptide expression of pooled data using Scaffold, and differential expression of proteins was determined by analysis with MetaboAnalyst. (**F**) A volcano plot of the 1830 proteins expressed by NDNs and LDNs from COVID-19 patients, comparing log_2_(fold change) to –log_10_(*P* value), with proteins above the red line demonstrating *P* < 0.05. A total of 326 proteins showed significantly greater expression in NDNs, and 134 proteins showed significantly greater expression in LDNs. (**G**) Differences in the pattern of protein expression by LDNs and NDNs were compared using orthogonal partial least squares discriminant analysis (orthoPLS-DA).

Previous studies showed that LDNs are markedly expanded in some COVID-19 patients (9), and we reported that a subset of LDNs with intermediate CD16 expression (CD16^int^) is associated with disease severity and worse clinical outcomes (9). Although the presence of CD16^int^ LDNs in BALF suggests they are recruited to the lungs in severe COVID-19, this LDN recruitment has not been directly demonstrated. Imaging mass cytometry analysis of lung tissue obtained from 5 COVID-19 patients (patient data in Table 1) at autopsy revealed extensive infiltration of LDNs (CD11b^+^CD66b^+^CD16^int^) into the lung parenchyma (Figure 1A). Additionally, the increased number of CD66b^+^CD15^+^CD16^int^ LDNs in peripheral blood of COVID-19 patients correlated with the disease severity (Figure 1B, patient data in Table 2). Therefore, we interpreted these data to indicate that a distinct LDN subset is recruited from the circulation into lung parenchyma in severe COVID-19.

To determine whether blood total LDN numbers return to normal levels after the acute phase, a separate cohort of convalescent COVID-19 patients were recruited at times ranging from 1 month to 13 months after hospital discharge (patient data in Table 3). We found that LDNs continued to be present in the peripheral blood, with higher numbers in convalescent patients than in age-matched healthy donors (Figure 1, C and D). In addition, LDN frequencies were lower with time from discharge (Figure 1E). Thus, persistently activated LDNs are present in convalescent patients, although their numbers wane over time, implying their potential contribution to PASC.

### LDNs from COVID-19 patients show distinct protein expression profiles compared with normal-density neutrophils that may contribute to more severe disease.

We previously reported that peripheral blood LDNs from severe COVID-19 patients show enhanced NET formation and cytokine production, but impaired degranulation and priming of phagocytosis and respiratory burst activity, compared with normal-density neutrophile (NDNs) (9, 15). LDNs from COVID-19 patients also showed enhanced interaction with platelets, while these platelets may be potently activated by CD16^int^ LDNs. To further establish differences between peripheral blood neutrophil subsets from COVID-19 patients, proteomic analysis (Figure 1, F and G) was performed on NDNs and LDNs isolated from 13 patients exhibiting increased LDNs from a second cohort of hospitalized COVID-19 patients. Following quantitation of peptides identified by mass spectrometry using Scaffold, a total of 1830 proteins were identified. As shown in a volcano plot (Figure 1F), 326 proteins exhibited significantly greater expression in NDNs, while 134 proteins exhibited greater expression in LDNs. The comparison of the protein expression pattern of LDNs and NDNs indicates distinctly different expression patterns by the 2 neutrophil subsets (Figure 1G). The 10 most significant Gene Ontology Biological Processes represented by proteins with significantly different expression between NDNs and LDNs are listed in Table 4. NDNs show enhanced expression of proteins involved in leukocyte activation and degranulation. On the other hand, proteins with increased expression by LDNs are involved in regulation of coagulation and complement activation. Analysis of these data revealed that CD16^int^ LDNs expressed higher levels of proteins corresponding to gene markers of immature neutrophils and lower levels of proteins associated with mature circulating neutrophils (Figure 1, F and G). These results are consistent with bulk RNA-seq data showing that CD16^int^ LDNs from COVID-19 patients exhibit higher expression levels of gene markers associated with immaturity compared with CD16^hi^ LDNs (9).

### Levels of plasma CXCL12 negatively correlated with percentage of peripheral blood LDNs.

**Figure 2:**
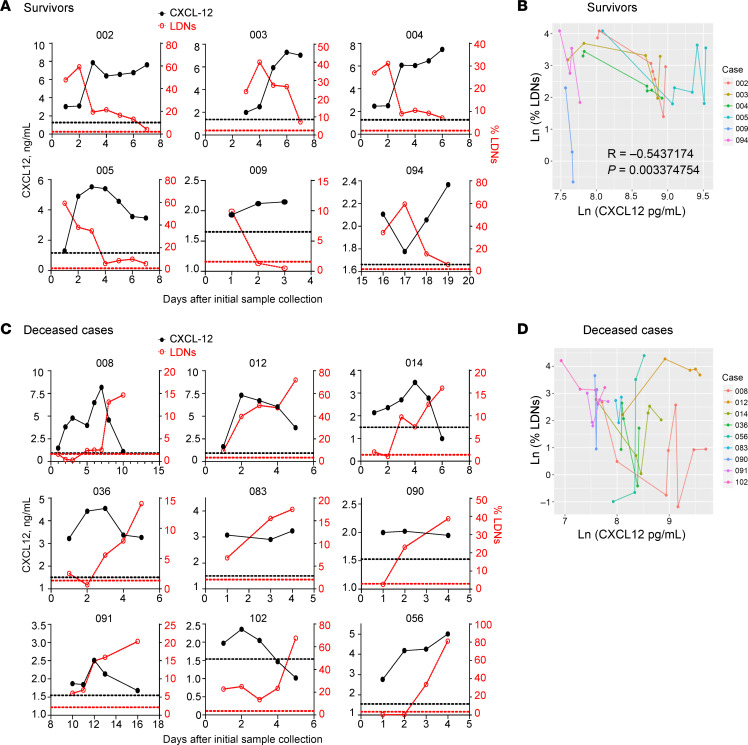
Plasma CXCL12 levels negatively correlate with peripheral blood LDNs in longitudinal analyses. (**A**) Peripheral blood samples from 6 SARS-CoV-2–infected survivors were collected longitudinally during hospitalization, as described previously (9). Concentrations of plasma CXCL12 and peripheral blood LDNs were measured. Black dotted line: Average plasma CXCL12 of healthy donors. Red dotted line: Average LDNs of healthy donors. (**B**) Correlation between concentration of plasma CXCL12 and percentage of peripheral blood LDNs analyzed by repeated measures correlation (with log transformation to Ln to meet linear assumption). *R* = –0.5437174 (*P* = 0.003374754). (**C**) Peripheral blood samples from 9 SARS-CoV-2–infected deceased patients were collected at multiple time points during hospitalization, as described previously (9). Concentrations of plasma CXCL12 and peripheral blood LDNs are shown. Black dotted line: Average CXCL12 of healthy donors. Red dotted line: Average LDNs of healthy donors. (**D**) Correlation between concentration of plasma CXCL12 and percentage of peripheral blood LDNs analyzed by repeated measures correlation (with log transformation to Ln to meet linear assumption). *R* = –0.01767992 (*P* = 0.9184839).

Although chemokines responsible for recruiting neutrophils to infected lungs, such as CXCL9, CXCL10, and CCL5, have been well studied (9, 36), whether some chemokines may provide negative feedback by regulating the distribution of inflammatory neutrophils remains poorly understood. The CXCR4/CXCL12 axis has important roles in the migration and distribution of neutrophils (37), including retention of immature neutrophils in the bone marrow prior to release into the circulation (38) and recruitment of apoptotic neutrophils into the bone marrow for destruction by macrophages (37–39). Because of these important roles for CXCL12 in neutrophil trafficking, we next assessed whether CXCL12 levels were related to changes in CD16^int^ LDN dynamics in SARS-CoV-2–infected patients, utilizing a previously described cohort of hospitalized COVID-19 patients (9). Serial peripheral blood samples collected at multiple time points after admission from 24 patients were used to determine the percentages of peripheral blood CD16^int^ LDNs by cytometry by time of flight (CyTOF) and plasma concentrations of CXCL12 by ELISA. In this cohort, 6 of 15 survivors showed a negative correlation between the percentages of peripheral blood CD16^int^ LDNs and plasma concentrations of CXCL12 during hospitalization (Figure 2, A and B). The other 9 survivors had neither increased numbers of blood LDNs (9) nor elevated CXCL12 levels (Supplemental Figure 1; supplemental material available online with this article; https://doi.org/10.1172/JCI188222DS1), making it impossible to carry out correlative analysis of the percentage of CD16^int^ LDNs and plasma CXCL12 levels. It is possible that these 9 patients were enrolled after their LDN levels peaked, during the period when CXCL12 levels had already declined. In contrast, all 9 deceased patients showed increased CD16^int^ LDNs, and the correlation between the percentage of CD16^int^ LDNs and CXCL12 levels was close to zero (*P* = 0.91) (Figure 2, C and D). These results suggest that the initial recruitment of LDNs was critical for inducing CXCL12, while the late accumulation of LDNs in deceased patients failed to trigger CXCL12 production. Together, these data are consistent with the notion that CXCL12 regulates CD16^int^ LDN accumulation in the circulation of COVID-19 survivors who have elevated numbers of CD16^int^ LDNs. As LDNs account for a substantial portion of neutrophils in the lung parenchyma, these findings support the conclusion that CXCL12 plays a regulatory role in COVID-19 immunopathogenesis.

### Neutrophilia contributes to disease severity of SARS2-N501Y_MA30_–infected mice.

**Figure 3:**
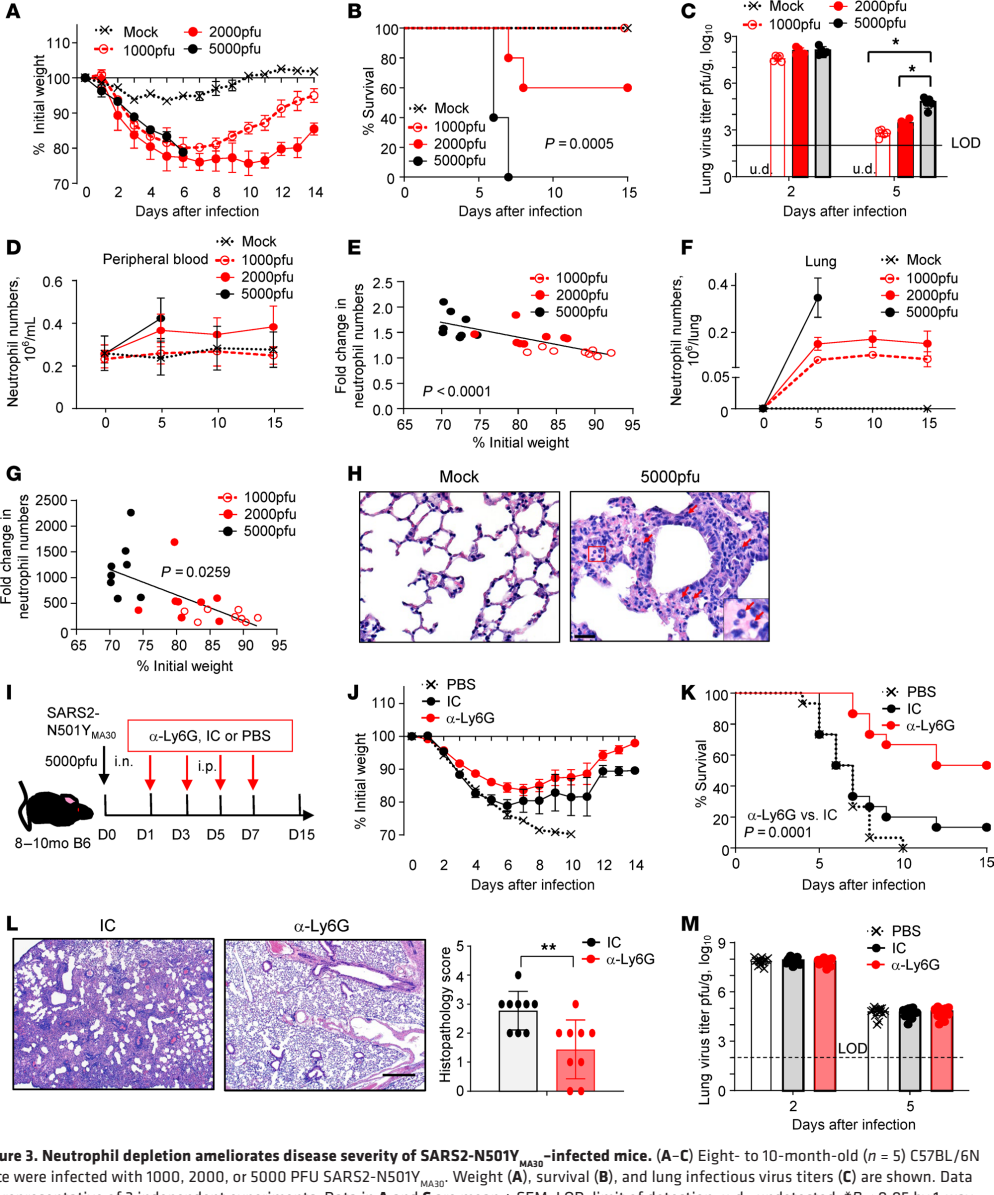
Neutrophil depletion ameliorates disease severity of SARS2-N501Y_MA30_–infected mice. (**A**–**C**) Eight- to 10-month-old (*n* = 5) C57BL/6N mice were infected with 1000, 2000, or 5000 PFU SARS2-N501Y_MA30_. Weight (**A**), survival (**B**), and lung infectious virus titers (**C**) are shown. Data are representative of 3 independent experiments. Data in **A** and **C** are mean ± SEM. LOD, limit of detection. u.d., undetected. *P < 0.05 by 1-way ANOVA with Tukey’s multiple comparisons in **C**. (**D**–**H**) Middle-aged C57BL/6N mice (8–10 months old, *n* = 5) were infected with 1000, 2000, or 5000 PFU SARS2-N501Y_MA30_ virus. (**D** and **F**) The number of neutrophils in peripheral blood (**D**) and lung (**F**) of infected (*n* = 8) and control mice (*n* = 5) was determined by flow cytometry at the indicated time points. Data are mean ± SEM and are representative of 3 independent experiments. (**E** and **G**) The correlation between the fold increase in peripheral blood (**E**) or lung-derived (**G**) neutrophils and weight change of SARS2-N501Y_MA30_–infected mice (*n* = 8) on day 5 after infection is shown. Data are representative of 3 independent experiments. (**H**) Infiltration of neutrophils (arrows) in lungs of mock- or SARS2-N501Y_MA30_–infected (5000 PFU) mice. Images are representative of 3 independent experiments. Arrows: PMNs. (**I**–**M**) Eight- to 10-month-old C57BL/6N mice were infected with 5000 PFU SARS2-N501Y_MA30_ and treated with PBS, α-Ly6G antibody, or isotype control (IC, isotype Ig) (*n* = 15 mice/group). Experimental setup (**I**), weight (**J**), survival (**K**), lung histopathology (**L**), and infectious virus titers (**M**) are shown. Data in **J** and **M** are mean ± SEM. Data in **K** are a summary of 3 independent experiments. Data in **L** are representative images and a summary of 2 independent experiments (data are mean ± SEM) (*n* = 9). **P < 0.01 by Student’s t-test. Scale bars: 25 μm (**H**) and 430 μm (**L**).

While these patient data are consistent with a role for LDNs in disease severity and for CXCL12 in protection, further mechanistic studies are difficult without a robust experimental model of COVID-19 infection. To develop such a system for studying acute and prolonged neutrophilia, we infected middle-aged (8- to 10-month-old) C57BL/6N mice intranasally with 1000, 2000, or 5000 PFU SARS2-N501Y_MA30_ to determine the optimal sublethal dose. Mice exhibited dose-related disease severity, characterized by weight loss, increased mortality, and elevated lung viral titers (Figure 3, A–C). In contrast, young (8- to 10-week-old) C57BL/6N mice did not succumb to challenge with the same doses of SARS2-N501Y_MA30_ (Supplemental Figure 2), mimicking the response observed in a majority of young SARS-CoV-2–infected patients.

Neutrophil activation/dysregulation, characterized by secreted NETs and proinflammatory cytokines/chemokines, is common in severe COVID-19 cases (9, 21, 40). Consistent with this human condition, numbers of neutrophils in peripheral blood (Figure 3, D and E) and lungs (Figure 3, F and G) in SARS2-N501Y_MA30_–infected mice (neutrophil gating strategies are shown in Supplemental Figure 3A) correlated with acute weight loss. Furthermore, neutrophils were recruited into the lung parenchyma of mice with severe COVID-19, analogous to the human infection (Figure 3H). In support of the pathogenic role of neutrophils in acute SARS2-N501Y_MA30_ infection, neutrophil depletion by α-Ly6G (α-Ly6G) antibody ameliorated weight loss and improved survival (Supplemental Figure 3, B and C, and Figure 3, I–L). The α-Ly6G treatment was therapeutic despite having no direct antiviral activity, as measured by lung virus titers (Figure 3M).

### LDN subset numbers correlate with infected-mouse disease severity.

**Figure 4:**
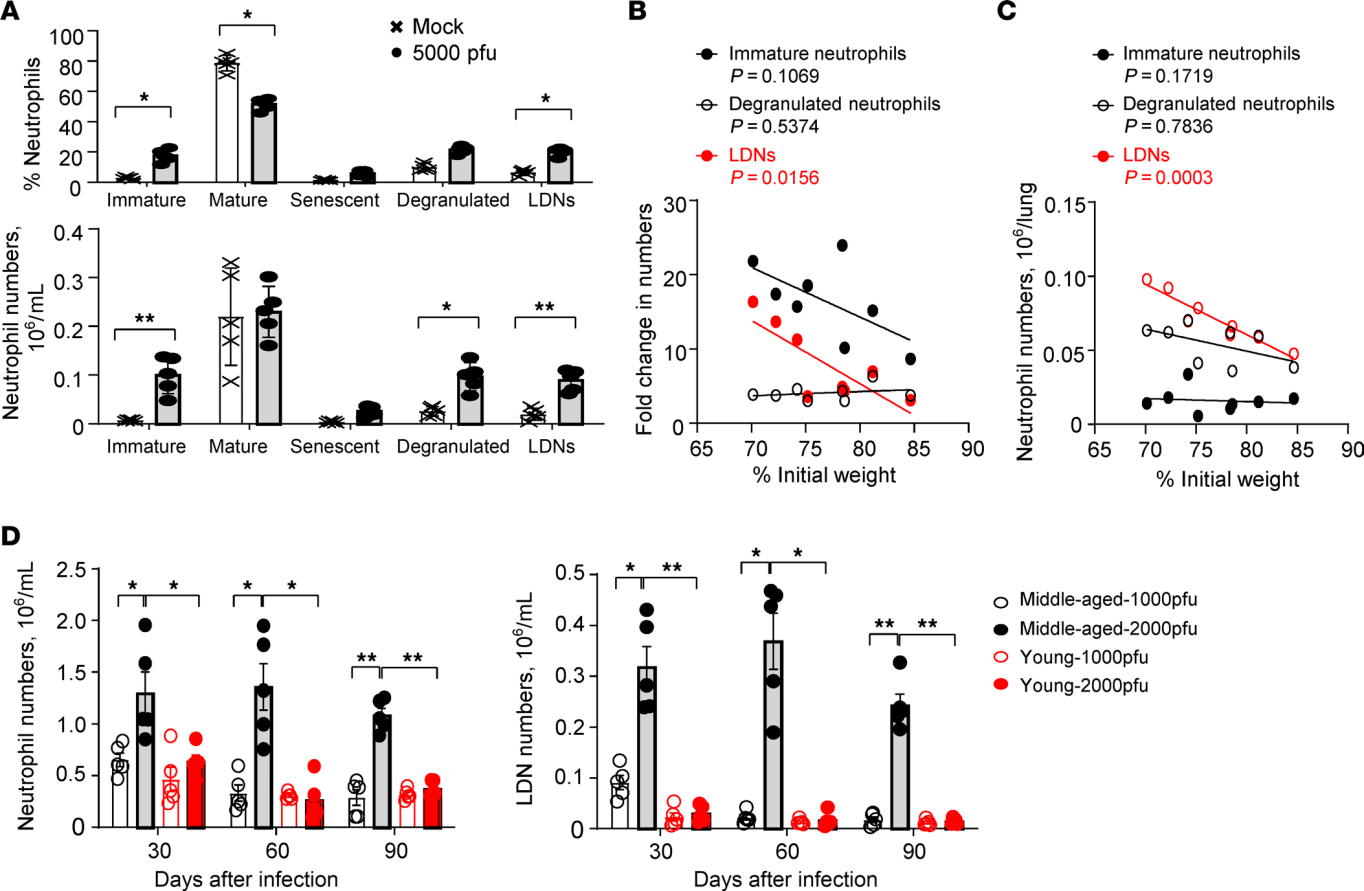
Accumulation of LDNs correlates with disease severity of SARS2-N501Y_MA30_–infected mice. (**A**) The percentage and absolute number of peripheral blood neutrophil subsets — CD15^+^CD16^+^CD115^–^CXCR2^–^ (immature), CD16^hi^CD62L^hi^CXCR2^hi^CXCR4^lo^ (mature), CD11b^hi^ CXCR2^lo^CD62L^lo^CXCR4^hi^ (senescent), CD11b^+^CD18^+^Gr-1^int^ (degranulated), and ARG1^+^CD15^+^CD33^+^CD101^–^CXCR4^+^ (LDNs) — in SARS2-N501Y_MA30_–infected mice on day 5 after infection (*n* = 5). Data are mean ± SEM and are representative of 3 independent experiments. *P < 0.05, **P < 0.01 by Student’s *t* test. (**B** and **C**) Correlation between fold increase in peripheral blood (**B**) and lung (**C**) immature neutrophils, degranulated neutrophils, and LDNs, and weight change of SARS2-N501Y_MA30_–infected mice on day 5 after infection (*n* = 8). Peripheral blood: *R* = 0.3744 (*P* = 0.1069), 0.06653 (*P* = 0.5374), and 0.6501 (*P* = 0.0156) for immature neutrophils, degranulated neutrophils, and LDNs. Lung: *R* =0.2862 (*P* = 0.1719), 0.01357 (*P* = 0.7836), and 0.9016 (*P* = 0.0003) for immature neutrophils, degranulated neutrophils, and LDNs. Data are representative of 2 independent experiments. (**D** and **E**) Young (8- to 10-week-old) or middle-aged (8- to 10-month-old) C57BL/6N mice were sublethally infected with 1000 or 2000 PFU. The numbers of peripheral blood neutrophils (**D**) and LDNs (**E**) were determined at the indicated time points by flow cytometry. *n* = 5. Data are mean ± SEM and are representative of 2 independent experiments. *P < 0.05; **P < 0.01 by 1-way ANOVA with Tukey’s multiple comparisons.

To determine the contribution of specific neutrophil subsets to the pathogenesis of acute COVID-19, flow cytometry was used to identify immature (CD15^+^CD16^+^CD115^–^CXCR2^–^), mature (CD16^hi^CD62L^hi^CXCR2^hi^CXCR4^lo^), senescent (CD11b^hi^CXCR2^lo^CD62L^lo^CXCR4^hi^), and degranulated (CD11b^+^CD18^+^Gr-1^int^) neutrophils, and LDNs (mouse LDNs are ARG1^+^CD15^+^CD33^+^CD101^–^CXCR4^+^) (41) in the peripheral blood of middle-aged mice infected with 5000 PFU of SARS2-N501Y_MA30_ (gating strategies are shown in Supplemental Figure 4A) (41). As summarized in Figure 4A, the percentage and the number of immature neutrophils and LDNs, but not mature neutrophils, in peripheral blood increased substantially in mice on day 5 after infection compared with mock-treated mice. Importantly, only the increased numbers of LDNs in peripheral blood (Figure 4B) and lungs (Figure 4C) correlated with weight loss. Furthermore, neutrophilia and increased LDNs persisted as long as 90 days after infection in middle-aged mice, long after mice recovered from severe disease (Figure 4, D and E). Thus, SARS-CoV-2 infection in mice recapitulated the increase in circulating LDNs and recruitment of LDNs into lung parenchyma observed in humans. Taken together, the amelioration of disease by neutrophil depletion in infected mice and the correlation of LDNs in the circulation and lungs with disease activity suggest that LDNs make a substantial contribution to the severity of COVID-19.

### CXCR4/CXCL12 regulates the accumulation of peripheral blood LDNs.

**Figure 5:**
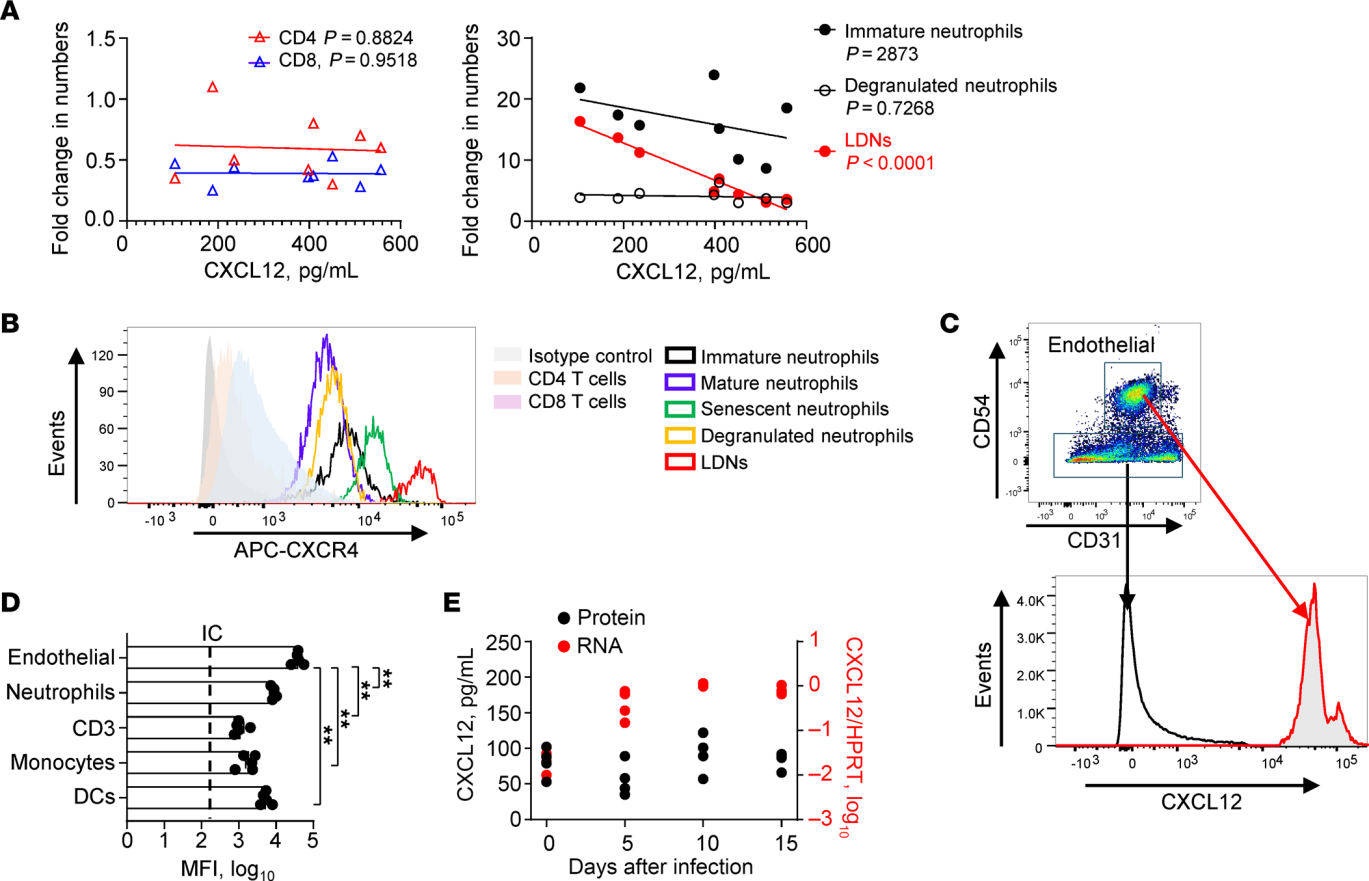
CXCL12/CXCR4 axis regulates blood neutrophil numbers in SARS2-N501Y_MA30_–infected mice. (**A**) The correlation between the concentration of plasma CXCL12 and the fold change in peripheral blood CD4^+^ and CD8^+^ T cells, immature neutrophils, degranulated neutrophils, and LDNs in SARS2-N501Y_MA30_–infected mice (5000 PFU) on day 5 after infection (*n* = 8). *R* = 0.003954 (*P* = 0.8824) (CD4^+^ T cells), 0.0006628 (*P* = 0.9518) (CD8^+^ T cells), 0.1851 (*P* = 0.2873) (immature neutrophils), 0.02186 (*P* = 0.7268) (degranulated neutrophils), and 0.9547 (*P* < 0.0001) (LDNs). Data are representative of 3 independent experiments. (**B**) Expression of CXCR4 by peripheral blood CD4^+^ and CD8^+^ T cells, and neutrophil subsets of mice infected with SARS2-N501Y_MA30_ on day 5 after infection. (**C**) Expression of intracellular CXCL12 in CD45^–^CD31^+^CD54^+^ vascular endothelial cells on day 5 after infection. (**D**) Summary of CXCL12 expression (mean fluorescence intensity, MFI) in peripheral blood cell subsets and endothelial cells, *n* = 5. Data are representative of 2 independent experiments and are mean ± SEM. **P < 0.01 by 1-way ANOVA with Tukey’s multiple comparisons. (**E**) RNA (right *y* axis) and protein (left *y* axis) CXCL12 levels in homogenates of bone marrow harvested from SARS2-N501Y_MA30_–infected mice were determined at the indicated time points by RT-qPCR and ELISA, respectively. *n* = 4. Data are representative of 2 independent experiments.

Since the clinical data in Figure 2 suggested an important role for CXCL12 in regulating LDNs in the blood and lungs, we further investigated factors important for neutrophil trafficking using SARS2-N501Y_MA30_–infected mice. We focused on chemokine/cytokine levels in the blood and lungs. In SARS-CoV-2–infected patients, several molecules were shown to be positively correlated with LDN numbers (CXCL10 in the BALF and IL-10, IL-1R, MCP-1, and MIP-1 in the plasma) (9). In contrast, CXCL12 was the sole chemokine in infected mice that correlated negatively with numbers of LDNs, while no chemokines were positively correlated (no significant associations were found between the levels of CXCL1, CXCL2, CXCL9, CXCL10, CCL2, CCL3, and CCL5 and LDN numbers). To further investigate the role of CXCL12 in LDN numbers, we measured plasma CXCL12 levels and assessed CXCR4 expression on neutrophils and CD4^+^ and CD8^+^ T cells in middle-aged mice infected with 5000 PFU of SARS2-N501Y_MA30_. Peripheral LDN numbers decreased as plasma CXCL12 levels increased, whereas no correlation was identified with other neutrophil subsets or CD4^+^/CD8^+^ T cells (Figure 5A). Compared with other neutrophil subsets and CD4^+^ and CD8^+^ T cells, LDNs expressed higher levels of CXCR4 (Figure 5B).

To identify the cellular origin of CXCL12, intracellular CXCL12 levels in peripheral blood and lung cell subsets, and in vascular endothelial cells (Figure 5, C and D, and Supplemental Figure 4B), were determined by flow cytometry. The highest levels of CXCL12 were detected in vascular endothelial cells and lung macrophages (Figure 5, C and D, and Supplemental Figure 4B). CXCL12 mRNA was significantly increased only in endothelial cells after infection, indicating that endothelial cells may be a source of plasma CXCL12 (Supplemental Figure 4C) and not just serve as “sinks” for circulating CXCL12. The increased CXCL12 in endothelial cells may keep circulating LDNs from infiltrating the lung parenchyma. As the bone marrow is a major source of CXCL12 in both homeostatic and pathological settings (42), CXCL12 RNA and protein expression in bone marrow from infected mice was measured by real-time quantitative polymerase chain reaction (RT-qPCR) and ELISA, respectively. CXCL12 mRNA in bone marrow homogenates increased after SARS2-N501Y_MA30_ infection, while protein levels of CXCL12 failed to increase (Figure 5E). The discrepancy between mRNA and protein levels of soluble factors is common and may be derived from diverse causes (43–45). Considering the increased plasma CXCL12, we speculate that the discrepancy in bone marrow CXCL12 mRNA and protein level could be due to its rapid binding to receptors or equilibration between bone marrow and blood. Thus, the bone marrow is likely also a site of CXCL12 production in infected mice, as previously reported (42). Together, these results suggest that CXCR4-CXCL12 interactions reduce lung infiltration by neutrophils by directing them to the vasculature and bone marrow, thereby diminishing the pathogenic effects of neutrophils.

### CXCL12 blockade enhances disease progression in SARS2-N501Y_MA30_–infected mice by regulating neutrophil distribution.

**Figure 6:**
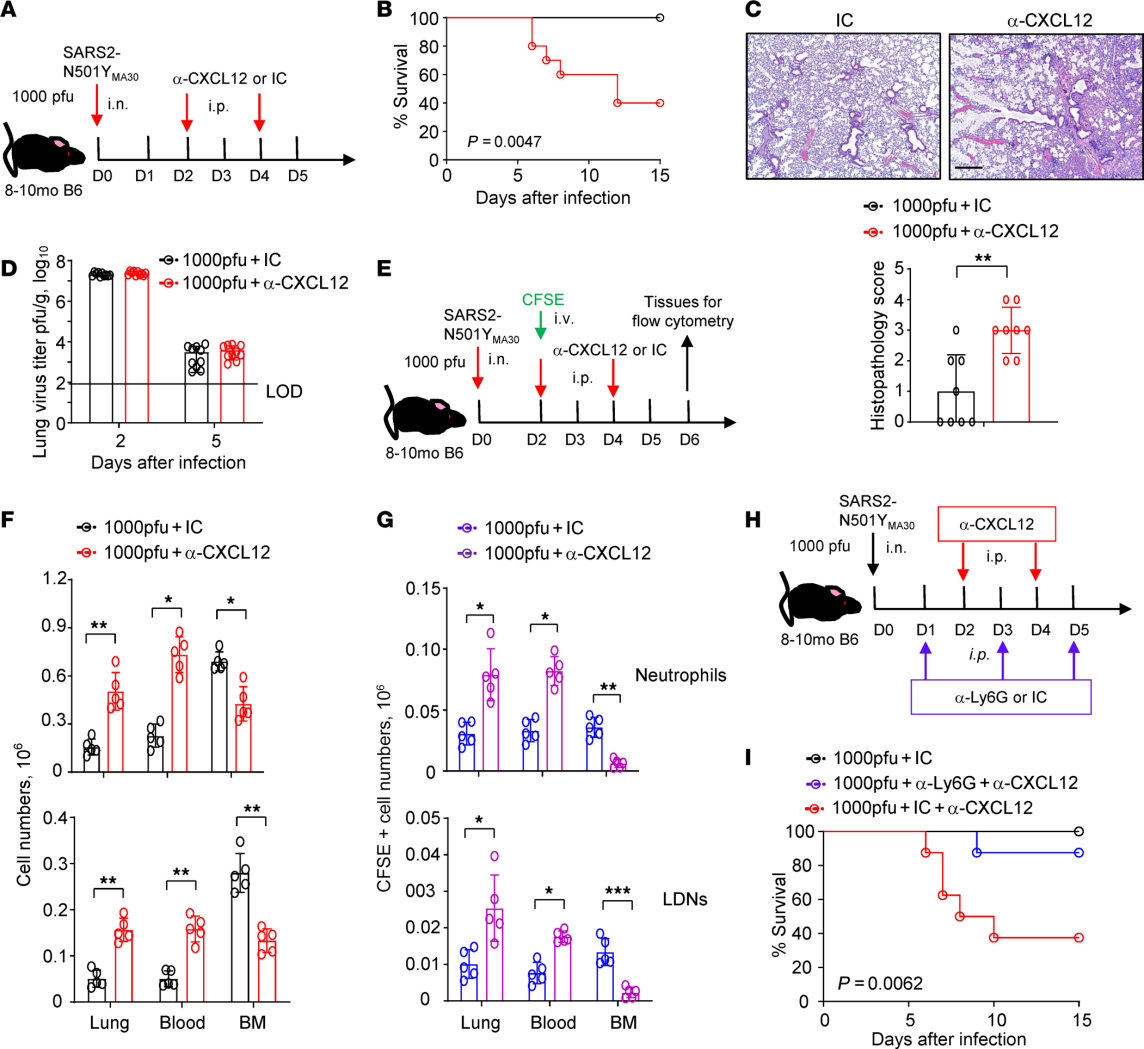
Blockade of CXCL12 modifies disease severity and neutrophil distribution. (**A**–**D**) Experimental setup (**A**), survival (**B**), lung histopathology (**C**), and infectious viral titers (**D**) of 8- to 10-month-old C57BL/6N mice infected with 1000 PFU SARS2-N501Y_MA30_ followed by treatment with α-CXCL12 antibody or its isotype control (IC, isotype Ig). Data in **B** are a summary of 4 independent experiments (*n* = 20). Data in **C** are representative images and a summary of 2 independent experiments (*n* = 10, samples harvested on day 5 after infection). Data in **D** are mean ± SEM (*n* = 8) and are a summary of 2 independent experiments. LOD, limit of detection. Scale bar: 430 μm. (**E**–**G**) Experimental setup (**E**), numbers of total neutrophils/LDNs (**F**), and CFSE-stained neutrophils/LDNs (**G**) identified in peripheral blood, lung, and bone marrow (BM) after treatment with α-CXCL12 antibody or IC (*n* = 5). Data are mean ± SEM and are representative of 2 independent experiments. *P < 0.05, **P < 0.01, ***P < 0.001 by t-test in **F** and **G**. (**H** and **I**) Experimental setup (**H**) and survival (**I**) of 8- to 10-month-old C57BL/6N mice infected with 1000 PFU SARS2-N501Y_MA30_ followed by treatment with α-CXCL12 antibody and α-Ly6G antibody or IC. Data in **I** are a summary of 2 independent experiments (*n* = 8).

To determine whether CXCL12 protects mice from disease progression, middle-aged C57BL/6N mice infected with a sublethal dose (1000 PFU) of SARS2-N501Y_MA30_ were treated with α-CXCL12 antibody or isotype control (Figure 6A). α-CXCL12 antibody treatment reduced CXCL12 levels in the blood, but did not diminish levels of CXCL12 protein in endothelial cells (Supplemental Figure 5, C and D), consistent with the notion that endothelial cells are a source of CXCL12 and do not function only to remove it from the blood. Furthermore, CXCL12 blockade increased mortality (Figure 6B) and lung histopathology scores (Figure 6C) of SARS2-N501Y_MA30_–infected mice, but did not change lung virus titers (Figure 6D).

Consistent with its pathogenic effects, α-CXCL12 antibody treatment (Figure 6E) resulted in increased accumulation of neutrophils in peripheral blood and lungs of infected mice (Figure 6F). This was likely due to reduced CXCL12-mediated sequestering of neutrophils in the bone marrow and, perhaps, enhanced neutrophil attachment to vascular endothelium. Next, we tracked peripheral neutrophil distribution after they were labeled intravenously with CFSE (Figure 6E). The numbers of CFSE^+^ neutrophils increased in the lung, but decreased in bone marrow after α-CXCL12 antibody treatment (Figure 6G), suggesting decreased recruitment back to the bone marrow (Figure 6F). To directly assess the role of neutrophils in α-CXCL12–enhanced disease, we treated SARS2-N501Y_MA30_–infected mice with α-CXCL12 antibody, in conjunction with α-Ly6G antibody or its isotype control (Figure 6H). Depletion of neutrophils rescued most mice from mortality caused by CXCL12 blockade (Figure 6I), consistent with a role for CXCL12 in ameliorating immunopathology by regulating the distribution of neutrophils, especially LDNs. Notably, at the dose of virus used in these experiments, no mice died. Therefore, neutrophil depletion would not be expected to improve outcomes in the absence of α-CXCL12 antibody treatment.

Of note, CXCL12 blockade had no effects on the numbers of neutrophil progenitor cells in the bone marrow (hematopoietic stem cells [HSCs], common myeloid precursors [CMPs], and granulocyte-monocyte progenitors [GMPs]) (Supplemental Figure 6, A and B). Finally, recombinant mouse CXCL12 administered intravenously modestly ameliorated disease severity (Supplemental Figure 6, C–E). Survival of mice was slightly prolonged, with differences trending toward statistical significance (Supplemental Figure 6E). Based on the results described above, effects on clinical disease would be expected to be greater if CXCL12 was targeted directly to the bone marrow or if a stabilized form was available (necessary because rodent CXCL12 has a half-life of approximately 26 minutes) (46).

### CXCL12 blockade does not affect adaptive immune responses in SARS2-N501Y_MA30_–infected mice.

CXCL12 is known to affect T cell trafficking (47), so we also examined whether CXCL12 blockade affected the generation of virus-specific neutralizing antibody or T cell responses (Supplemental Figure 7A). Virus-specific T cell responses were measured directly ex vivo by stimulating cells with SARS-CoV-2 peptide pools (S protein, membrane protein, and nucleoprotein peptide pools). As shown in Supplemental Figure 7, B–D, no significant differences were found in numbers of total T cells or levels of neutralizing antibody in mice receiving α-CXCL12 antibody or control treatment. Moreover, CXCL12 blockade did not affect the development of virus-specific memory CD4^+^ and CD8^+^ T cell responses in the blood or lungs when assessed on day 30 after SARS2-N501Y_MA30_ infection (Supplemental Figure 7, E and F). CXCL12 treatment increased the numbers of blood neutrophils and LDNs, but did not affect the distribution of T cell subsets in naive mice (Supplemental Figure 7, G and H). These results are not unexpected because CXCR4 levels were much lower on T cells than neutrophils (Figure 5B) and there was no correlation between CXCL12 concentration and T cell numbers (Figure 5A). To further confirm that T cells were relatively unresponsive to modulation of the CXCR4/CXCL12 axis, we treated middle-aged mice with extremely high doses of α-CXCL12 antibody (100 mg/kg). As shown in Supplemental Figure 7, I and J, high doses of α-CXCL12 antibody modestly decreased the number of CD4^+^ and CD8^+^ T cells in naive mice, but had no effect in SARS-CoV-2–infected mice. Moreover, to confirm the specificity of the α-CXCL12 blocking antibody, we assessed its binding to CXCL12 protein. As shown in Supplemental Figure 8, the blocking antibody and the antibody used in ELISA bound the CXCL12 protein equivalently. These data collectively indicated that T cell migration is slightly altered upon CXCL12 blockade, but much higher antibody doses were required compared with the level needed to affect neutrophil trafficking.

### CXCL12 expression by endothelial cells is SARS-CoV-2 specific and involves ACE2 binding.

**Figure 7:**
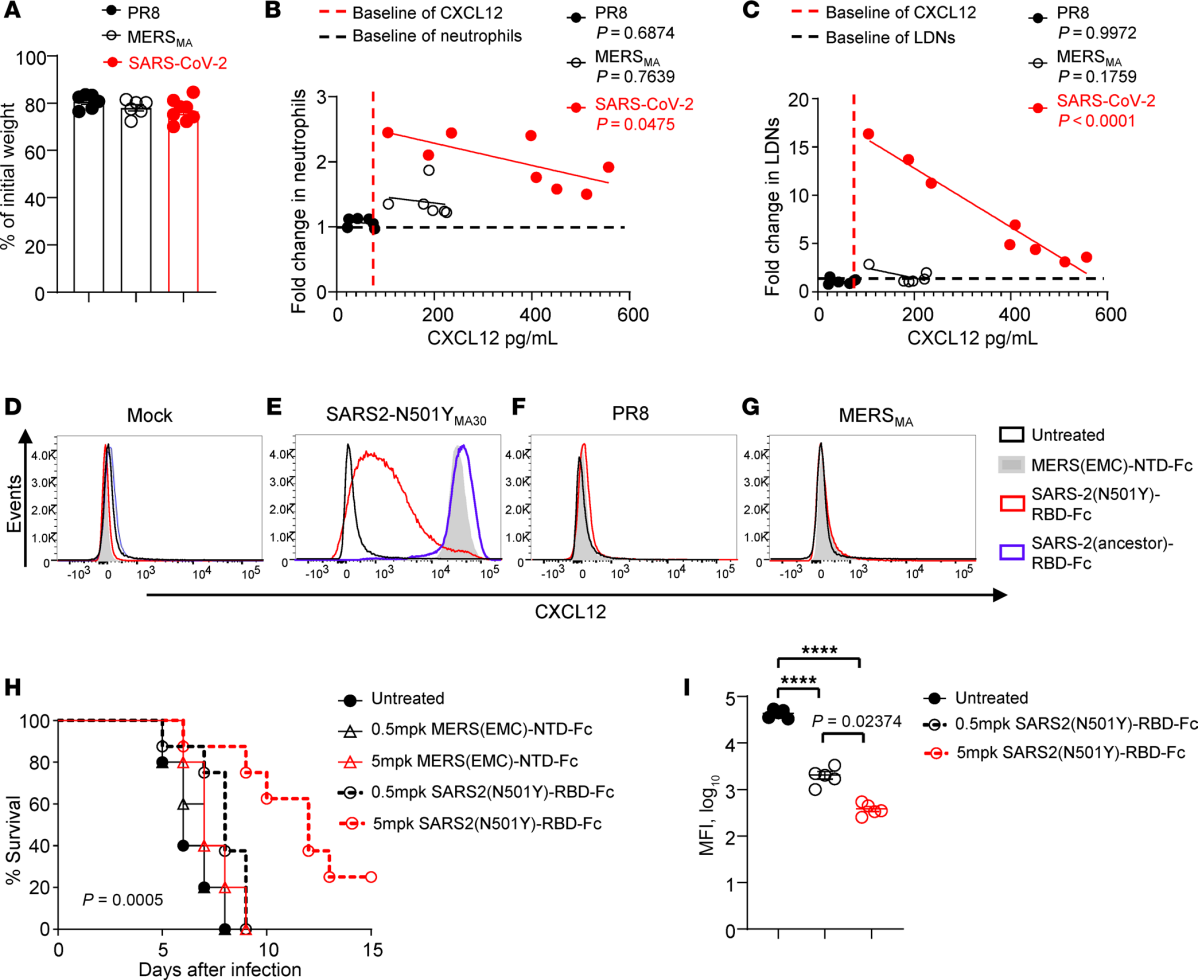
SARS-CoV-2-RBD-Fc modifies CXCL12 expression by vascular endothelial cells and the outcome of SARS-CoV-2 infection. (**A**) Weights of mice infected with 500 PFU IAV-PR8 (*n* = 6), 500 PFU MERS_MA_ (*n* = 6), or 5000 PFU SARS2-N501Y_MA30_ (*n* = 8) measured on day 5 after infection. (**B** and **C**) Correlation between plasma CXCL12 concentration and fold change in peripheral blood neutrophils (**B**) and LDNs (**C**) in IAV-PR8–, MERS_MA_-, and SARS2-N501Y_MA30_–infected mice on day 5 after infection. (**B**) *R* = 0.04474 (*P* = 0.6874) (IAV-PR8), 0.02521 (*P* = 0.7639) (MERS_MA_), and 0.5072 (*P* = 0.0475) (SARS2-N501Y_MA30_). (**C**) *R* = 3.492 × 10^–6^ (*P* = 0.9972) (IAV-PR8), 0.4028 (*P* = 0.1759) (MERS_MA_), and 0.9547 (*P* < 0.0001) (SARS2-N501Y_MA30_). Data are representative of 2 independent experiments. Mock- (**D**), SARS2-N501Y_MA30_–infected (5000 PFU) (**E**), or IAV-PR8–infected (500 PFU) (**F**) middle-aged C57BL/6N mice (8–10 months old, *n* = 5/group), or MERS_MA_-infected (500 PFU) *hDPP4*-KI mice (**G**) were treated with 0.5 mg/kg body weight of MERS (EMC)-NTD-Fc, SARS-2 (N501Y)-RBD-Fc, or SARS-2 (ancestral)-RBD-Fc in 0.5 mL PBS by i.v. injection on days 2 and 4 after infection. Mice were euthanized on day 5 after infection and abdominal aortas were harvested. The expression of CXCL12 in endothelial cells was determined by intracellular staining via flow cytometry. Data are representative of 2 independent experiments. (**H** and **I**) SARS2-N501Y_MA30_–infected (5000 PFU) mice were treated with SARS2 (N501Y)-RBD-Fc (*n* = 8) or control MERS (EMC)-NTD-Fc (*n* = 5). Survival (**H**) and endothelial cell expression of CXCL12 (**I**) were determined. ****P < 0.01 by 1-way ANOVA with Tukey’s multiple comparisons. Data are mean ± SEM and are representative of 2 independent experiments. mpk, mg/kg body weight.

Endothelial cell CXCL12 mRNA and protein upregulation by SARS-CoV-2 infection was unexpected, so we next assessed whether this upregulation was a generalized response to respiratory virus infection. We infected mice with SARS2-N501Y_MA30_ or 2 other mouse-adapted pathogenic human respiratory viruses, influenza A virus (IAV-PR8) and mouse-adapted Middle East respiratory syndrome–CoV (MERS_MA_) (48) with virus doses that resulted in equivalent clinical disease (Figure 7A). SARS-CoV-2, but not IAV-PR8 or MERS_MA_, infection induced significantly increased numbers of neutrophils and LDNs in the blood (Figure 7, B and C). Meanwhile, CXCL12 expression in endothelial cells was increased only after infection with SARS-CoV-2 (Figure 7, D–G). This result raised the possibility that SARS-CoV-2 interactions with ACE2 on endothelial cells (48) was critical for increased CXCL12 expression by these cells. To assess this possibility, we engineered the SARS-CoV-2 receptor binding domain (RBD) conjugated to a stabilizing immunoglobulin Fc (SARS-2-RBD-Fc), and as a control, MERS-CoV (EMC/2012) S protein N-terminal domain–Fc (MERS-CoV S NTD-Fc). As shown in Figure 7, E, H, and I, treatment with MERS-CoV S NTD-Fc or SARS-2-RBD-Fc from ancestral strains of SARS-CoV-2, which cannot bind to mACE2, had no effect, whereas treatment with low amounts of SARS-2 (N501Y) RBD-Fc, which can bind to mACE2, resulted in CXCL12 downregulation in endothelial cells. Of note, only high amounts of SARS-2 (N501Y) RBD-Fc prolonged survival, probably by more effectively blocking virus entry into susceptible cells.

## Discussion

Here we show that neutrophilia and increased levels of LDNs, characteristic manifestations of severe COVID-19, are also observed in mice infected with SARS-CoV-2. Of note, we observed the benefits of neutrophil depletion in mice with severe disease, which is in contrast with a previous report showing no effect of depletion in mice with mild disease (49), suggesting a role for neutrophils mainly in severe disease. Consistent with their increased number in peripheral blood (9), LDNs represent the major neutrophil population accumulating in the lung parenchyma of patients with severe COVID-19 (Figure 1A). In addition, LDNs expressed increased levels of proteins that are associated with complement and coagulation cascades (Figure 1, F and G, and Table 4), suggesting a critical role of LDNs in SARS-CoV-2–related inflammation and thrombosis. More importantly, consistent with observations made on patient samples (9), numbers of LDNs were increased in SARS-CoV-2–infected mice and their number correlated with clinical disease severity (Figure 4, B and C).

Plasma CXCL12, the ligand for CXCR4, is upregulated in COVID-19 (50), and has been shown to be a marker for disease severity (51). Polymorphisms in CXCL12 were shown to correlate with disease severity, emphasizing the importance of this chemokine (52). CXCR4 expression on LDNs (Figure 5B) enhanced their migration to sites of CXCL12 production. Our results further demonstrate the protective role of CXCL12 in SARS-CoV-2 infection in mice (Figure 6, B–D). Notably, the negative correlation in the blood and lungs between LDN numbers and CXCL12 expression (Figure 4C and Figure 5A) was also observed in COVID-19 survivors (Figure 2, A and B). To our best knowledge, no other chemokine or cytokine levels negatively correlated with LDN numbers to the same extent in patient plasma, although other molecules such as GM-CSF are upregulated in patients with severe COVID-19 and could contribute to neutrophilia (53).

Strikingly, upregulation of CXCL12 expression by endothelial cells is observed specifically in murine infection with SARS-CoV-2, but not after influenza A virus or MERS-CoV infection (Figure 7, E–G). Endothelial cells are known to express ACE2 (54), and endothelial dysfunction is a well-described characteristic of SARS-CoV-2 infection (55). While endothelial cells do not appear to be productively infected by SARS-CoV-2, infectious virus is required for increased CXCL12 expression; SARS-2 (N501Y) RBD-Fc by itself does not result in enhanced CXCL12 expression (Figure 7D). CXCL12 expression by endothelial cells could result from direct interactions with the virus, or indirectly through virus-ACE2 interactions on other cells, with subsequent effects on endothelial cells. SARS-CoV-2 binding to ACE2 may interfere with normal ACE2 function, resulting in activation of NF-κB and increased production of proinflammatory molecules (56). These data suggest that, in addition to limiting neutrophil egress from the bone marrow (57), CXCL12-CXCR4 interactions protect against lung injury in SARS-CoV-2 infections by regulating neutrophil trafficking into the lungs. These effects on trafficking are abrogated after treatment with α-CXCL12 antibody (Figure 6, F and G), resulting in increased numbers of LDNs in the lungs and clinical deterioration. Further work will be required to determine the relative importance of CXCL12 expression by bone marrow versus endothelial cells in regulating the dynamics of LDNs and other immune cells. In addition, monocytes, dendritic cells (DCs), and neutrophils also expressed CXCL12, albeit at substantially lower levels than endothelial cells (Figure 5D), which could also contribute to elevated CXCL12 levels in the blood. Although CXCL12 is expressed by alveolar epithelial cells (58), increased LDN recruitment into the lungs after CXCL12 blockade suggests that LDNs migrate to the lung in response to other chemoattractants, such as CXCL1, CCL2, and CXCL10, expressed in the infected lung (36). It is also noteworthy that lung-recruited neutrophils were also found to contribute to the pathogenesis of influenza A virus infection (59, 60). Although IAV-PR8 infection failed to increase blood neutrophil numbers or CXCL12 expression by endothelial cells (Figure 7, B–D), the involvement of the CXCL12/CXCR4 axis in regulating neutrophil, especially LDN, accumulation in the lungs and bone marrow deserves further investigation.

Additionally, we show that neutrophilia persists in mice that survive SARS-CoV-2 infection (Figure 4, D and E). Long-term effects of SARS-CoV-2, including lung fibrosis, neuroinflammation, and behavioral changes, are apparent in previously infected hamsters and mice (61–63), and neutrophilia could contribute to these sequelae. These results mirror clinical observations that neutrophilia persists for several months in patients after resolution of acute SARS-CoV-2 infection (64) (Figure 1, C–E). Increased expression of mRNAs and proteins associated with neutrophil-mediated inflammation was detected in COVID-19 patients with persistent lung changes at 3–12 months after infection (25). Changes in markers indicative of increased NET formation were identified in these patients. Extracellular newly identified receptor for advanced glycation end-products binding protein (EN-RAGE), expressed by LDNs in COVID-19 patients (18), and the chemokine IL-17C, important for neutrophil migration (65), were also expressed at higher levels in COVID-19 survivors with interstitial/fibrotic pulmonary disease. LDN numbers correlated with more severe disease in patients with acute COVID-19 (9, 18, 21), as well as in other conditions, including sepsis (66). In most settings, LDNs are proinflammatory and have decreased chemotactic ability, decreased phagocytic activity, and increased expression of proinflammatory molecules, similar to the LDNs that we identified (Figure 1, F and G, and Table 4).

There are limitations with these studies. First, it will be critical to specifically deplete LDNs in mice and assess effects on clinical disease. No useful antibody or other method for depleting LDNs is now available, making these experiments impossible. Second, our data show that CXCL12 had important effects on neutrophils, particularly LDN trafficking in SARS-CoV-2 infections. However, in the absence of biomarkers specific for LDNs, the role of CXCL12/CXCR4 signaling in LDN trafficking is not fully proven. Meanwhile, CXCR12/CXCR4 signaling appears to have different roles in different clinical settings and is difficult to precisely define, partly because CXCL12-null mice are not viable (67, 68). Thus, CXCL12 blockade or conditional CXCL12 knockout in tumors (69, 70) and neuroinflammatory disease models (71–73) has led to contradictory conclusions, suggesting that the role of CXCL12/CXCR4 signaling is disease context dependent. Given the pleiotropic effects of CXCL12, it will also be important to extend these analyses to studies of the role of CXCL12 in trafficking of other immune cells such as NK cells and plasmacytoid DCs in infected mice.

Collectively, our results show that neutrophilia can persist for extended periods of time after resolution of acute SARS-CoV-2 in infected mice and patients. Decreases in LDN numbers, which contribute to disease resolution in mice, are dependent on interactions between CXCL12 and CXCR4. These data support the value of measuring CXCL12 levels to predict disease severity and long-term sequelae of COVID-19 infections and identify it as a possible target for therapeutic intervention.

## Methods

### Sex as a biological variable.

In preliminary experiments, we did not observe significant differences between male and female mice. Consequently, equal numbers of male and female mice were used in these studies.

### Study participants and clinical data.

Inclusion criteria were all hospitalized adults (older than 18) who had positive SARS-CoV-2 results and consented to this study. Exclusion criteria included age younger than 18 or refusal to participate. Patients enrolled in this study were diagnosed with a RT-qPCR–based 2019-CoV detection kit at the University of Louisville hospital laboratory using nasopharyngeal swab samples obtained from patients. All patients with acute SARS-CoV-2 infection were followed by the research team daily, and the clinical team was blinded to findings of the research analysis to avoid potential bias. Demographic characteristics (age, sex, height, weight, BMI, and clinical data: symptoms, comorbidities, laboratory findings, treatments, complications, and outcomes) were collected prospectively. The infected patients described in Figure 2 were previously reported (9).

### Virus.

SARS2-N501Y_MA30_ and MERS_MA_ were generated as described previously (32, 74). Mouse-adapted IAV A/PR/8/34 (IAV-PR8) was a gift from K. Legge (Department of Pathology, University of Iowa).

### Mice.

Eight- to 10-week-old or 8- to 10-month-old male and female C57BL/6N mice were obtained from Charles River Laboratories. *hDPP4*-KI mice were developed and propagated as described previously (74). Mice were maintained in the Animal Care Unit at the University of Iowa and Regional Biocontainment Laboratory (RBL) at the University of Louisville under standard conditions of dark/light cycle, ambient temperature, and humidity. Mice were randomly assigned to different groups, with numbers per group sufficient to obtain statistical significance.

### Mouse infection.

In most experiments, mice were infected lethally (5000 PFU) or sublethally (1000 or 2000 PFU) with SARS2-N501Y_MA30_. Some mice were infected sublethally with 500 PFU influenza A virus PR8 (IAV-PR8) or 500 PFU mouse-adapted MERS-CoV (MERS_MA_). Animal weight and health were monitored daily. All experiments with SARS-CoV-2 and MERS_MA_ were performed in a Biosafety Level 3 (BSL3) laboratory at the University of Iowa and the University of Louisville. Experiments with IAV-PR8 were performed in a BSL2 Laboratory at the University of Louisville.

### Plasma and PBMC isolation.

Whole blood samples of patients were centrifuged at 541*g* for 10 minutes. Plasma and PBMCs were processed as described previously (9).

### Human lung slides preparation and imaging mass cytometry.

Lung tissue sections from deceased COVID-19 patients were stained with metal-labeled antibodies: α-CD66b (BLR11H)-166Er (catalog 91H033166, 1:150); α-CD16 (EPR16784)-149Sm (catalog 91H004149, 1:150); α-collagen (polyclonal)-142Nd (catalog 91H018142, 1:300); α-αSMA (1A4)-153Eu (catalog 91H001153, 1:300); and α-pan-cytokeratin (AE-1/AE3)-174Yb (catalog 91H006174, 1:200) were purchased from Standard BioTools. Purified α-CD15 (W6D3) (BioLegend, catalog 323035) and α-SARS-CoV-2 Spike S1 subunit (R&D Systems, catalog MAB105407) were labeled with 172Yb and 173Yb at 1:100 and 1:200 dilutions, respectively (Maxpar X8 Multimetal Labeling Kit, catalog 201300, Standard BioTools). All antibodies were first validated to confirm optimal staining intensity, specificity, and signal-to-noise ratio. Stained tissue sections were ablated by using the Hyperion mass cytometry imaging system (Standard BioTools). The Hyperion was autotuned using a 3-element tuning slide (Standard BioTools) as described in the Hyperion imaging system user guide. An extra minimum threshold of 1000 mean duals of ^175^Lu was used. At least three 2500 × 1500 μm regions of interest (ROIs) per sample were selected and ablated at 200 Hz. Data were visualized by using MCD viewer software (Standard BioTools). For downstream analysis, image data were exported as tiff.ome files from the MCD viewer, followed by cell segmentation using CellProfiler (developed by Broad Institute of MIT and Harvard, v4.2.1; https://cellprofiler.org/); fcs files or cvs files were exported using histoCAT software. The fcs files were further analyzed using FlowJo software (BD).

### Synthesis of Fc-recombinant protein.

SARS-2-RBD-Fc and a control construct, MERS (EMC)-NTD-Fc, were synthesized and evaluated previously (75). MERS (EMC)-NTD-Fc contains the MERS-CoV S protein NTD bound to Fc. SARS-2 (N501Y)-RBD-Fc was synthesized following site-directed mutagenesis of the N501 codon within pCEP4-SARS-2-RBD-Fc. The pCEP4 expression plasmids were transfected into HEK293T cells using LipoD (SignaGen, catalog SL100668). Transfected cells were grown in FBS-free DMEM containing 2% (wt/vol) Cell Boost 5 (HyClone, catalog SH30865.01). Conditioned media were collected on days 3 and 6 and clarified free of debris (300*g*, 4°C, 10 minutes; 4,500*g*, 4°C, 10 minutes). Fc-tagged proteins were purified using HiTrap protein A high-performance columns (GE Healthcare, catalog GE17-0402-01) according to the manufacturer’s instructions. Purified proteins were dialyzed in PBS (pH 7.4), quantified spectrophotometrically, and stored at −20°C until use.

### Histopathology and scoring.

Formalin-fixed, paraffin-embedded lungs were sectioned (~4 μm) and stained with hematoxylin and eosin (H&E). Tissues were evaluated by a board-certified pathologist using the postexamination method of masking to groups (76). Edema was ordinarily scored (0–4) as previously described (32).

### Collection of whole blood/serum from mice.

Mice were anesthetized by intraperitoneal injection of ketamine-xylazine. Blood was collected through retro-orbital bleed with a capillary tube (Thermo Fisher Scientific). Blood was allowed to clot at room temperature for 30 minutes. Sera were clarified by centrifugation and transferred to a new tube for storage at –80°C. For collection of whole blood, heparinized capillary tubes were used (Thermo Fisher Scientific).

### Lung, spleen, bone marrow, and abdominal aorta cell preparation.

After perfusion, lungs and abdominal aorta of mice were removed, minced, and digested in HBSS buffer consisting of 2% fetal calf serum, 25 mM HEPES, 1 mg/mL collagenase D (Roche), and 0.1 mg/mL DNase (Roche) at 37°C for 30 minutes. To collect bone marrow, the ends of femurs were cut and the marrow plug was flushed with 1 mL of complete RPMI 1640 media. Single-cell suspensions of lungs, spleens, bone marrow, and abdominal aortas were prepared by passage through a 70 μm cell strainer. Lung macrophages (CD45^+^CD68^+^F4/80^+^) and vascular endothelial cells (CD45^–^CD31^+^CD54^+^) were purified from single-cell suspensions of lungs and abdominal aortas using a BD FACSAria.

### Flow cytometry.

Cells were enumerated with a Scepter 2.0 cell counter (MilliporeSigma), washed and blocked with 1 μg α-CD16/α-CD32 antibodies (clone 2.4G2, StemCell) at 4°C for 20 minutes, and surface stained with the following antibodies at 4°C for 30 minutes: APC α-mouse CD16/CD32 (clone 2.4G2, BD BioScience), V450 α-mouse CD45 (clone 30-F11), APC α-mouse B220 (clone RA3-6B2), APC/Cyanine 7 α-mouse CD3e (clone 145-2C11), APC/Cyanine 7 α-mouse CD11c (clone FC), FITC or PE or BV21 α-mouse Ly6G (clone 1A8), BV510 or PE α-mouse CD11b (clone M1/70), PE/Cyanine 7 α-mouse CD8 (clone 53-6.7), PerCP/Cyanine 5.5 α-mouse CD4 (clone RM4-5), PerCP/Cyanine 5.5 α-mouse Ly6C (clone HK1.4), PE α-mouse CD 64 (clone X54-5/7.1), BV421 α-mouse CD115 (clone AFS98), Alexa Fluor 488 α-mouse CD15 (clone MC480), APC α-mouse CD18 (clone H155-78), APC α-mouse CD31 (clone W18222B), APC α-mouse CD33 (clone W18124D), PE α-mouse CD34 (clone HM34), FITC α-mouse CD54 (clone YN1/1.7.4), Pacific Blue α-mouse CD62L (clone W18021D), PE-Cy7 α-mouse Gr-1 (clone RB6-8C5), PE-Cy7 α-mouse CD68 (clone FA11), PE-Cy7 α-mouse CD93 (clone AA4.1), PE-Cy7 α-mouse CD101 (clone Moushi101, Invitrogen), FITC α-mouse CD127 (clone SB/199), BV421 α-mouse CD135 (clone A2F10), PE-Cy5.5 α-mouse CXCR2 (clone SA045E1), FITC or PE-Cy5.5 α-mouse CXCR4 (clone L276F12), FITC α-mouse MHC-II (clone 39-10-8), APC α-mouse ARG1 (R&D Systems), Pacific Blue α-mouse F4/80 (clone BM8), BV510 α-mouse Sca-1 (clone D7), and APC-Cy7 α-mouse c-kit (clone ACK2). Antibodies were purchased from BioLegend if not otherwise specified and were used at 1:200 dilution. Cells were fixed and permeabilized with Cytofix/Cytoperm (BD Biosciences).

For intracellular cytokine staining, lymphocytes were cultured in 96-well dishes at 37°C for 5–6 hours in the presence of 2 μM peptide pools and brefeldin A (BD Biosciences), labeled for cell-surface markers, fixed/permeabilized with Cytofix/Cytoperm Solution (BD Biosciences), and labeled with PE α-mouse CXCL12 (clone MAB310, R&D Systems), APC α-mouse IFN-γ (clone XMG1.2, BioLegend), and FITC α-mouse TNF (clone MP6-XT22, BioLegend) antibodies (1:100 dilution). All flow cytometry data were acquired using a BD FACSVerse and analyzed with FlowJo software.

### RNA isolation and RT-qPCR.

Total RNA was extracted from tissues using TRIzol (Invitrogen) or a Direct-zol RNA Miniprep kit (Zymo Research) according to the manufacturer’s protocol. cDNA was prepared and the primers used for cytokine and chemokines were previously reported (65). For detection of CXCL12, the following primers were used: F: 5′-GGAGGATAGATGTGCTCTGGAAC-3′ and R: 5′-AGTGAGGATGGAGACCGTGGTG-3′.

### ELISA.

Concentrations of human and mouse plasma CXCL12 were determined by ELISA (Human CXCL12/SDF-1α Quantikine ELISA Kit, DSA00; Mouse CXCL12/SDF-1α Quantikine ELISA Kit, MCX120, R&D Systems) according to the manufacturer’s instructions.

The binding capability of α-CXCL12 antibodies (clone MAB310 and MCX120) was determined by ELISA using reagents and standard samples included in the ELISA Kit (MCX120, R&D Systems).

### CFSE staining and cell tracking.

To track the migration of peripheral blood neutrophils, 8- to 10-month-old C57BL/6N mice were infected with SARS2-N501Y_MA30_ (1000 or 2000 PFU) and treated with 2 mg/kg CFSE (Invitrogen) diluted in 0.2 mL PBS on day 2 after infection via tail vein injection. CFSE^+^ cells in peripheral blood, lung, and bone marrow were determined by flow cytometry at the indicated time points.

### Treatment with α-Ly6G, α-CXCL12, and rCXCL12.

For neutrophil depletion, infected mice were treated on days 1, 3, 5, and 7 after infection with 0.2 mL PBS or 20 mg/kg α-Ly6G (clone 1A8, Bio X Cell) or its isotype control Ig (rat IgG2a, clone 2A3, Bio X Cell) diluted in 0.2 mL PBS. For α-CXCL12 treatment, mice were treated on days 2 and 4 after infection with 0.2 mL PBS, or 25 or 100 mg/kg α-CXCL12 (clone MAB310, R&D Systems) or its isotype Ig (mouse IgG1, clone MAB002, R&D Systems). For rCXCL12 treatment, mice were treated with 10 mg/kg rCXCL12 (R&D Systems) diluted in 0.2 mL PBS on days 2, 5, and 8 after infections. Drugs and PBS were administered via tail vein injection.

### Whole human blood analysis.

For whole blood analysis, 150 μL of whole blood was lysed with 2 mL of ACK buffer for 10 minutes. Cells were spun down and washed once with PBS. Cells were then stained with APC-Cy7 viability dye, PeCy7 α-human CD45 (clone 2D1), PE α-human CD66b (clone 6/40c), and APC α-human CD16 (clone 3G8, all from BioLegend) for 30 minutes at 4°C prior to washing and analysis using a FACSCanto (BD Biosciences).

### CyTOF mass cytometry sample preparation.

As described previously (9), mass cytometry antibodies were either purchased preconjugated (Standard BioTools) or were conjugated in house using MaxPar X8 polymer kits or MCP9 polymer kits (Standard BioTools) according to the manufacturer’s instructions. PBMCs were isolated as described above, stained for viability with 5 μM cisplatin (Standard BioTools), washed, and stained with the complete antibody panel for 30 minutes at room temperature. Cells were fixed in 1.6% formaldehyde for 10 minutes at room temperature, and then incubated overnight in 125 nM Intercalator-Ir (Standard BioTools) at 4°C. Cells were washed twice with cell staining buffer (Standard BioTools) and then resuspended at a concentration of 1 million cells/mL in cell acquisition solution containing a 1:9 dilution of EQ 4 Element Beads (Standard BioTools). The samples were acquired on a Helios (Standard BioTools) at an event rate of less than 500 events/s. After acquisition, the data were normalized using bead-based normalization in the CyTOF software and gated to exclude residual normalization beads, debris, dead cells, and doublets, leaving DNA^+^CD45^+^Cisplatin^lo^ events for subsequent clustering and high-dimensional analyses.

### Virus titer by plaque assay.

Virus or tissue homogenate supernatants were serially diluted in DMEM and titered on VeroE6 (for SARS-CoV-2), Vero81 (for MERS-CoV), or MDCK (for IAV-PR8) cells, as previously described.

### CyTOF data analysis.

CyTOF data were analyzed using a combination of the Cytobank software package (77) and the CyTOF workflow (78), which consists of a suite of packages (79) available in R (https://www.r-project.org/). For analysis conducted within the CyTOF workflow, FlowJo Workspace files were imported and parsed using functions within flowWorkspace and CytoML. An arcsinh transformation (cofactor = 5) was applied to the data using the dataPrep function within CATALYST and stored as a “singlecellexperiment” object. Cell population clustering and visualization were conducted using FlowSOM (80) and ConsensusClusterPlus (79) within the CyTOF workflow and using the viSNE application within Cytobank. Clustering was performed using data across all donors and time points. Additionally, clustering was performed either using all live CD45^+^ cells or after gating on CD66b^+^ neutrophils.

### Analysis of human neutrophil proteomes.

NDNs and LDNs were isolated from whole blood by plasma Percoll gradients, followed by magnetic bead purification, as previously described (81). For mass spectrometry, cells were disrupted by sonication, followed by protein extraction with 2% sodium dodecyl sulfate. Protein extracts were digested using an S-trap micro spin column (Protifi, LLC) digestion protocol. For proteomic analyses, a Dionex Ultimate 3000 RSLCnano system (Thermo Fisher Scientific) was used to inject the digests (250 ng) onto a 300 μm × 5 mm, 5 μm PepMap Neo C18 trap cartridge heated at 30°C (Thermo Fisher Scientific). The trapped peptides were then resolved using a 75 μm × 15 cm, 3 μm, 100 Å PepMap RSLC C18 EASY-spray separating column heated at 40°C with a 90-minute 5%–35% acetonitrile gradient accomplished at 200 nL. An EASY-spray source (Thermo Fisher Scientific) was used to control ion transfer into the mass spectrometer at 320°C and 1.8 kV. An Orbitrap Exploris 480 mass spectrometer (Thermo Fisher Scientific) was used to collect data from the LC eluate. A full MS-ddMS2 method with a 3-second cycle time was created in Xcalibur v4.5.445.18 (Thermo Fisher Scientific) operating in positive polarity. Scan event 1 of the methods obtained an MS1 scan (60,000 resolution, normalized AGC target of 100%, scan range 350–1400 *m*/*z*). Scan event 2 obtained dd-MS2 scans (7,500 resolution, normalized AGC target of 50%) on ions with charge states from 2–6 and a minimum intensity of 8,000 until the cycle time was complete.

Proteome Discoverer v2.5.0.400 (Thermo Fisher Scientific) was used to analyze the data collected by the mass spectrometer. In the processing step, the database used in SequestHT was the July 17, 2023 version of the UniprotKB-reviewed canonical *Homo* *sapiens* sequences (Proteome ID UP000005640). Trypsin (KR|P) digestion with up to 2 missed cleavages was assumed with the dynamic modifications Oxidation (M), Acetyl (Protein N-term), Met-los (Protein N-term), and Met-loss+Acetyl (Protein N-term); and the static modification Carbamidomethyl (C). Precursor, and fragment mass tolerances were 10 ppm and 0.02 Da, respectively. In the consensus step, proteins were quantified from the summed abundances of all high-confidence unique and razor peptide intensities. Samples were normalized to total peptide amount and scaled to 100%. Proteins were grouped by the strict parsimony principle. Peptides and proteins were accepted at 1% FDR for high confidence or 5% for medium confidence based on the *q* value. A proteins text file was exported from the consensus workflow result of Proteome Discoverer for curation in Microsoft Excel.

Data from differentially expressed proteins were analyzed by MetaboAnalyst (v5.0) (https://genap.metaboanalyst.ca/). Partial least squares discrimination analysis of differential protein expression among the cell groups was created to establish differences among the groups. A correlation analysis of proteins differentially expressed in the neutrophil populations was plotted. Analysis of differentially expressed proteins was performed using Gene Ontology Enrichment Analysis (https://geneontology.org/docs/go-enrichment-analysis/). Protein-protein interaction network analysis was performed using Search Tool for the Retrieval of Interacting Genes/Proteins (STRING v10) (https://string-db.org/),with the highest confidence score (0.900).

### Statistics.

Differences between group means were analyzed by 1-way ANOVA (with Tukey’s multiple comparisons) and 2-tailed Student’s *t* tests, and differences in time-to-death were analyzed by log-rank (Mantel-Cox) tests using Microsoft Excel and GraphPad Prism 8. All results are expressed as mean ± SEM and were corrected for multiple comparisons. The association between human peripheral blood LDNs and days after discharge, the association between mouse neutrophil numbers, and weight change (Figure 1E, Figure 3, E and G, and Figure 4, B and C), and the association between mouse neutrophil numbers and plasma CXCL12 (Figure 5A and Figure 7, B and C) were analyzed by simple linear regression. The relationship between patient plasma CXCL12 and the frequency of LDNs (Figure 2, B and D) was analyzed via repeated measures correlation. A *P* value of less than 0.05 was considered statistically significant.

### Study approval.

Approval for using human samples was obtained from the IRB at the University of Louisville. Written informed consent was obtained from either participants or their legal authorized representatives (IRB 20. 0321). All animal studies were approved by the University of Iowa and University of Louisville Animal Care and Use Committees and met stipulations of the NIH *Guide for the Care and Use of Laboratory Animals* (National Academies Press, 2011).

### Data availability.

The data supporting the findings of this study are documented within the paper and are available from the corresponding authors upon request. Raw data underlying the figures are available in the Supporting Data Values file. Correspondence and requests for materials should be addressed to: Stanley Perlman (stanley-perlman@uiowa.edu) or Jian Zheng (jian.zheng.1@louisville.edu).

## Author contributions

JZ designed the study and experiments, collected data, and contributed to data interpretation and manuscript preparation. HD, EQ, RS, AEG, SMM, DS, XH, Hong Li, Haiyan Li, and DM contributed to data collection and interpretation. KW and KK contributed to study design. LHW and PTE contributed to data statistical analysis and interpretation. PSH, WJJ, and RB contributed to obtaining permission to biobank lung samples from patients, storing samples, analyzing the histopathology, and providing clinical data on the patients. DKM contributed to histopathology and scoring. KP and SMU contributed to data interpretation and manuscript preparation. MLM contributed to data generation and data analysis in the proteomic analysis. KM, TG, JH, and JY designed experiments and contributed to data interpretation and manuscript preparation. SP designed and coordinated the study, designed experiments and contributed to data interpretation, data presentation, and manuscript preparation.

## Supplementary Material

Supplemental data

Supporting data values

## Figures and Tables

**Table 4:**
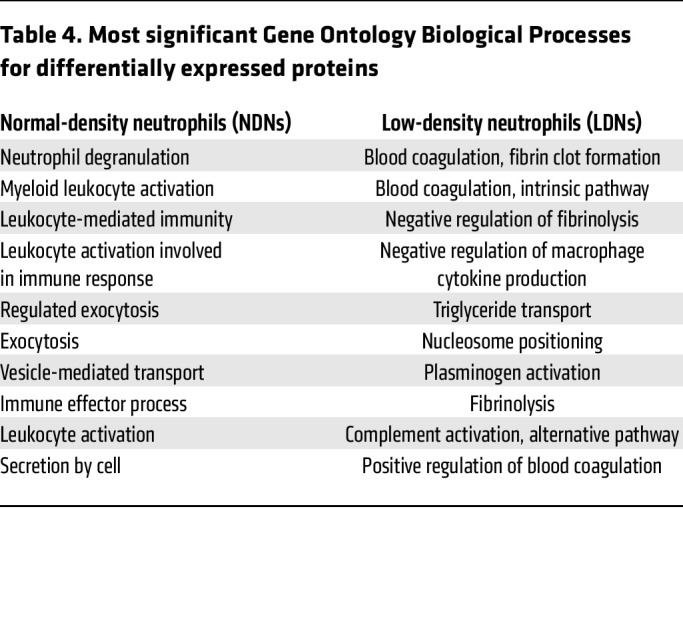
Most significant Gene Ontology Biological Processes for differentially expressed proteins

**Table 3:**
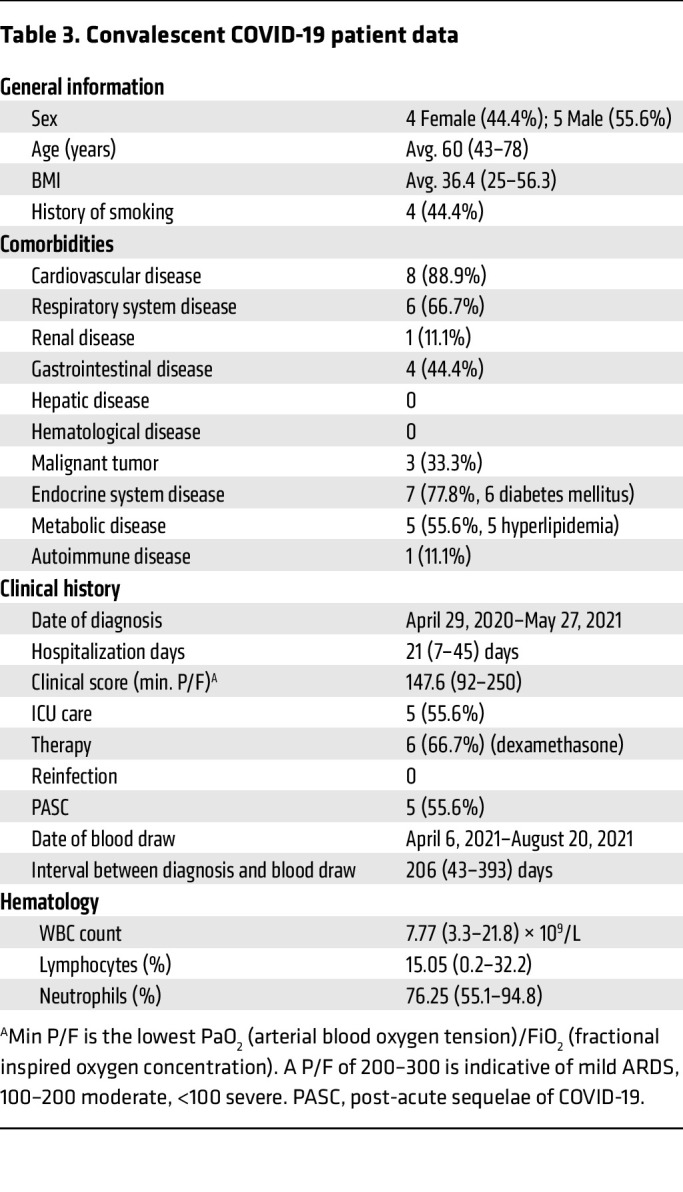
Convalescent COVID-19 patient data

**Table 2:**
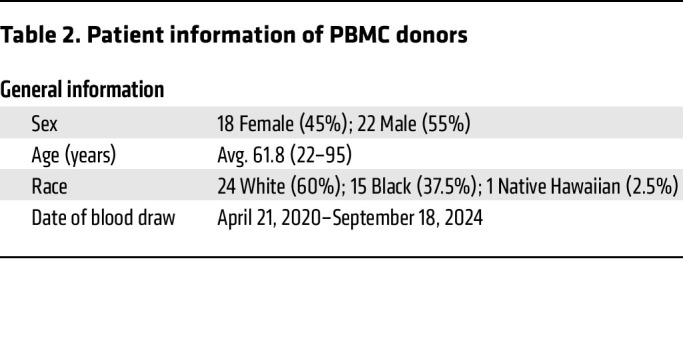
Patient information of PBMC donors

**Table 1:**
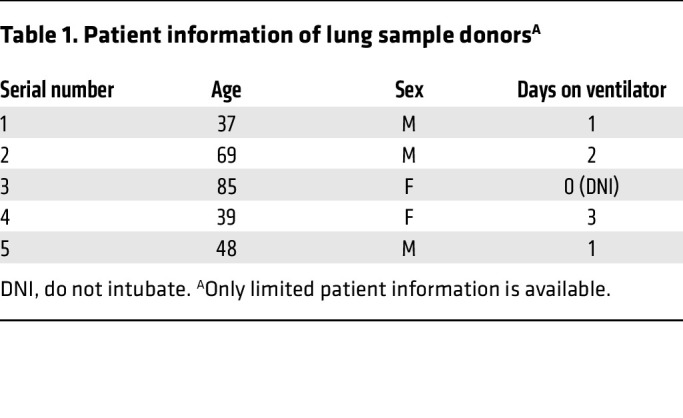
Patient information of lung sample donors^A^

## Version 1 01/07/2025

In-Press Preview

## Version 2 02/17/2025

Electronic publication
