# Supplementary material for: CXCL12 ameliorates neutrophilia and disease severity in SARS-CoV-2 infection
Source: J Clin Invest. 2025 Jan 7;135(4):e188222. doi: 10.1172/JCI188222 (PMC11827850; doi:10.1172/JCI188222)
Supplement: Supplemental data [file jci-135-188222-s201.pdf]

CXCL12 ameliorates neutrophilia and disease severity in SARS-CoV-2 infection

Supplemental Figure legends

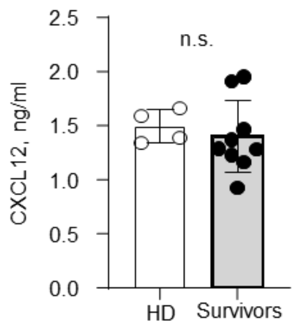

Supplemental Figure 1. Plasma CXCL12 in COVID-19 survivors. Concentrations of plasma CXCL12 in 9 SARS-CoV-2-infected survivors showing no increased LDNs and 4 healthy donors (HD). n.s., not significant.

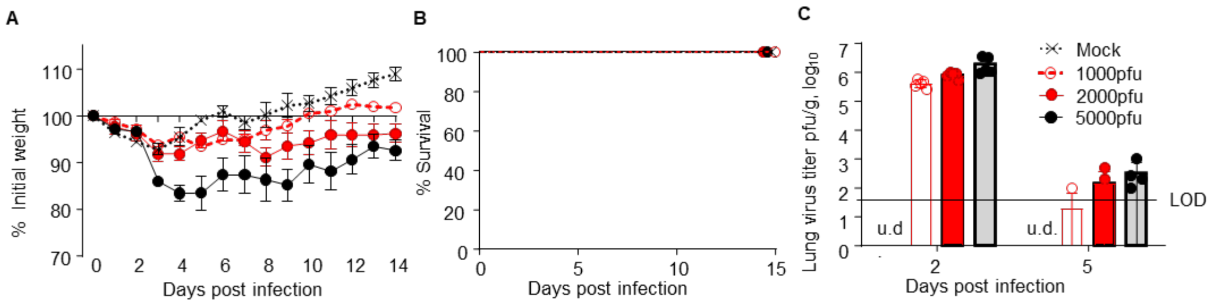

Supplemental Figure 2. Young mice show resistance to SARS2-N501Y$_{MA30}$ infection. 8-10-week-old (n=5, **A-C**) C57BL/6N mice were infected with 1000, 2000 or 5000 pfu SARS2-N501Y$_{MA30}$. Weight (**A**), survival (**B**) and lung infectious virus titers (**C**) are shown. Data are representative of two independent experiments, respectively. Data in **A** and **C** are mean±SEM. LOD, limit of detection. u.d., undetected. ANOVA F-test $P<0.05$ in (**C**).

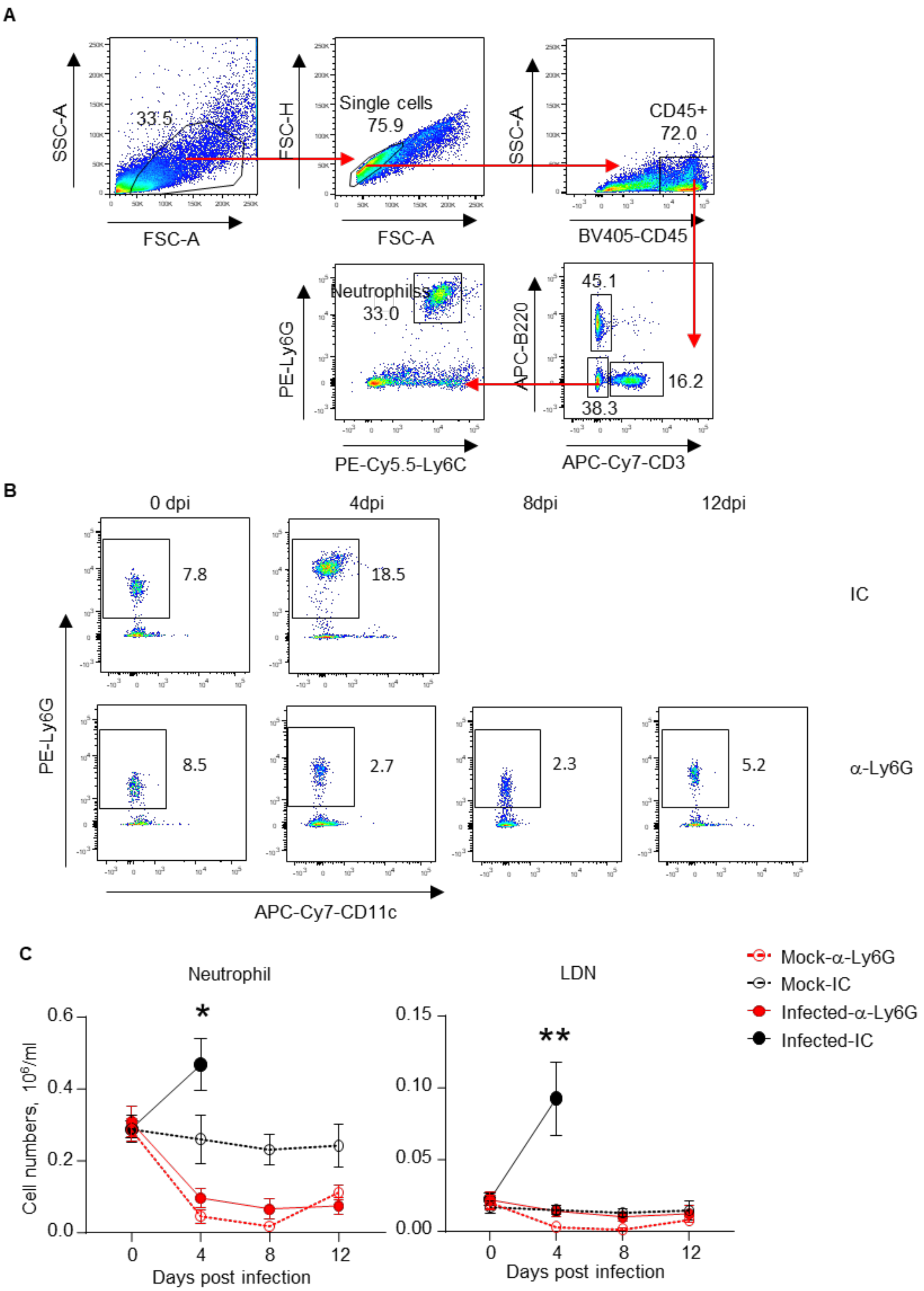

Supplemental Figure 3. Efficacy of neutrophil depletion. (**A**) Gating strategy for identification of mouse neutrophils. Neutrophils are CD45$^+$CD3$^-$B220$^-$Ly6G$^+$Ly6C^{low}. (**B** and **C**) 8-10-month-old C57BL/6N mice were mock infected or infected with 5000 pfu SARS2-N501Y$_{MA30}$, followed by anti-Ly6G antibody or isotype control (IC, isotype Ig) treatment at 1, 3, 5, 7 dpi. The frequency

of peripheral blood neutrophils was determined by flow cytometry at indicated time points. (**B**) Representative flow cytometric data obtained from a mouse infected with 5000 pfu SARS2-N501Y$_{MA30}$ and treated with anti-Ly6G or IC. Summary data (**C**) are mean±SEM and are representative of two independent experiments (n= 5). *P< 0.05 by t-test (Infected-anti-Ly6G vs. Infected-IC).

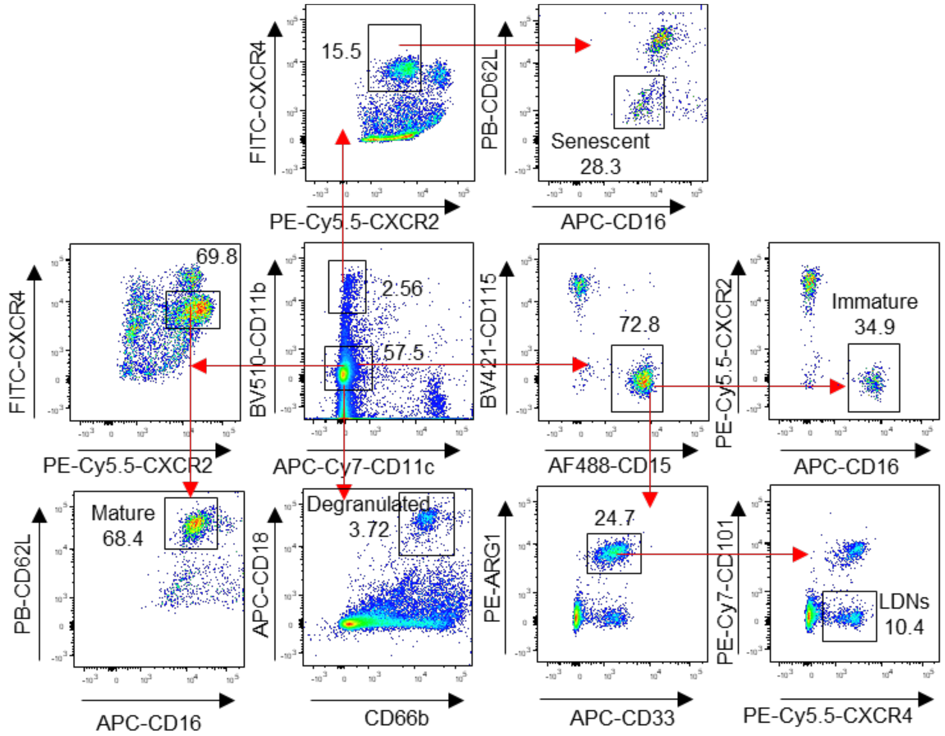

Supplemental Figure 4. Gating strategy for identification of neutrophil subsets. Neutrophil subsets were identified by flow cytometry in 8-10-month C57BL/6N mice infected with SARS2-N501Y$_{MA30}$ at 5 days post infection.

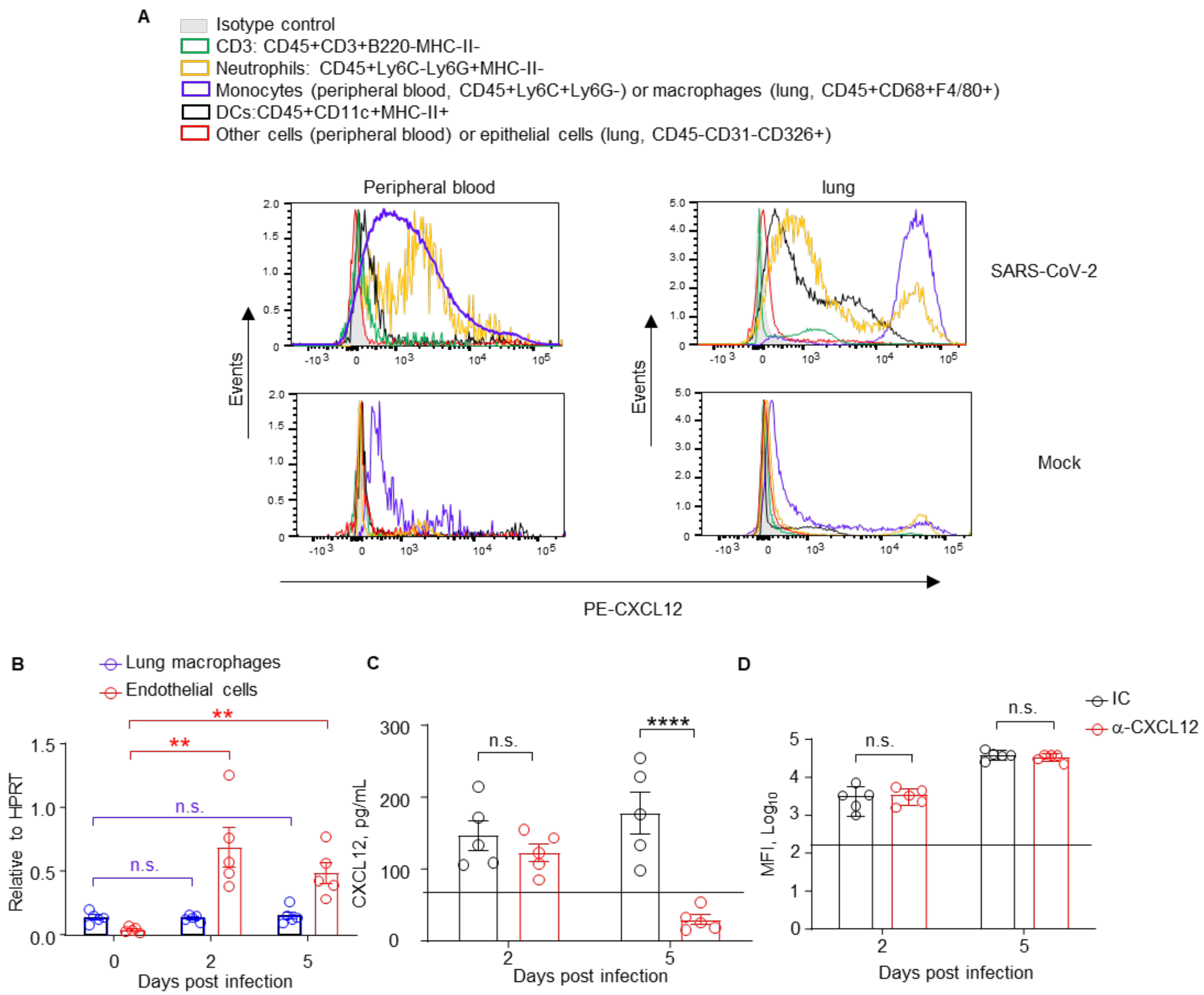

Supplemental Figure 5. Expression of CXCL12 in immune and endothelial cells. (**A**) Expression of intracellular CXCL12 in immune cell subsets (CD45$^+$CD3$^+$CD19$^-$MHC-II$^-$ T lymphocytes; CD45$^+$Ly6C$^-$Ly6G$^+$MHC-II$^-$ neutrophils; CD45$^+$Ly6C$^+$Ly6G$^-$ monocytes; CD45$^+$ CD68$^+$F4/80$^+$ lung macrophages; CD45$^+$CD11c$^+$MHC-II$^+$ DC; CD45$^-$CD31$^-$CD326$^+$ lung epithelial cells and other cells in peripheral blood). Cells used for flow cytometry were harvested from peripheral blood and lungs of mock- or SARS2-N501Y$_{MA30}$-infected mice at day 5. Data are representative of three independent experiments. (**B**) Expression of CXCL12 mRNA in lung macrophages and vascular endothelial cells of SARS2-N501Y$_{MA30}$-infected mice (5000 pfu) at indicated time points. n=5. Data are representative of two independent experiments and are mean±SEM. n.s., not significant. **P< 0.01 by ANOVA F-test. (**C** and **D**) 8-10-month-old C57BL/6N mice were infected with 1000 pfu SARS2-N501Y$_{MA30}$ and treated with anti-CXCL12 antibody or its isotype control (IC, isotype Ig) at days 3 and 5 post infection. Summary data of

CXCL12 levels in peripheral blood (ELISA, **C**) and CXCL12 expression in endothelial cells (RT-PCR, **D**) are shown. n=5. Data are representative of two independent experiments and are mean±SEM. n.s., not significant. ****P< 0.0001 by ANOVA F-test.

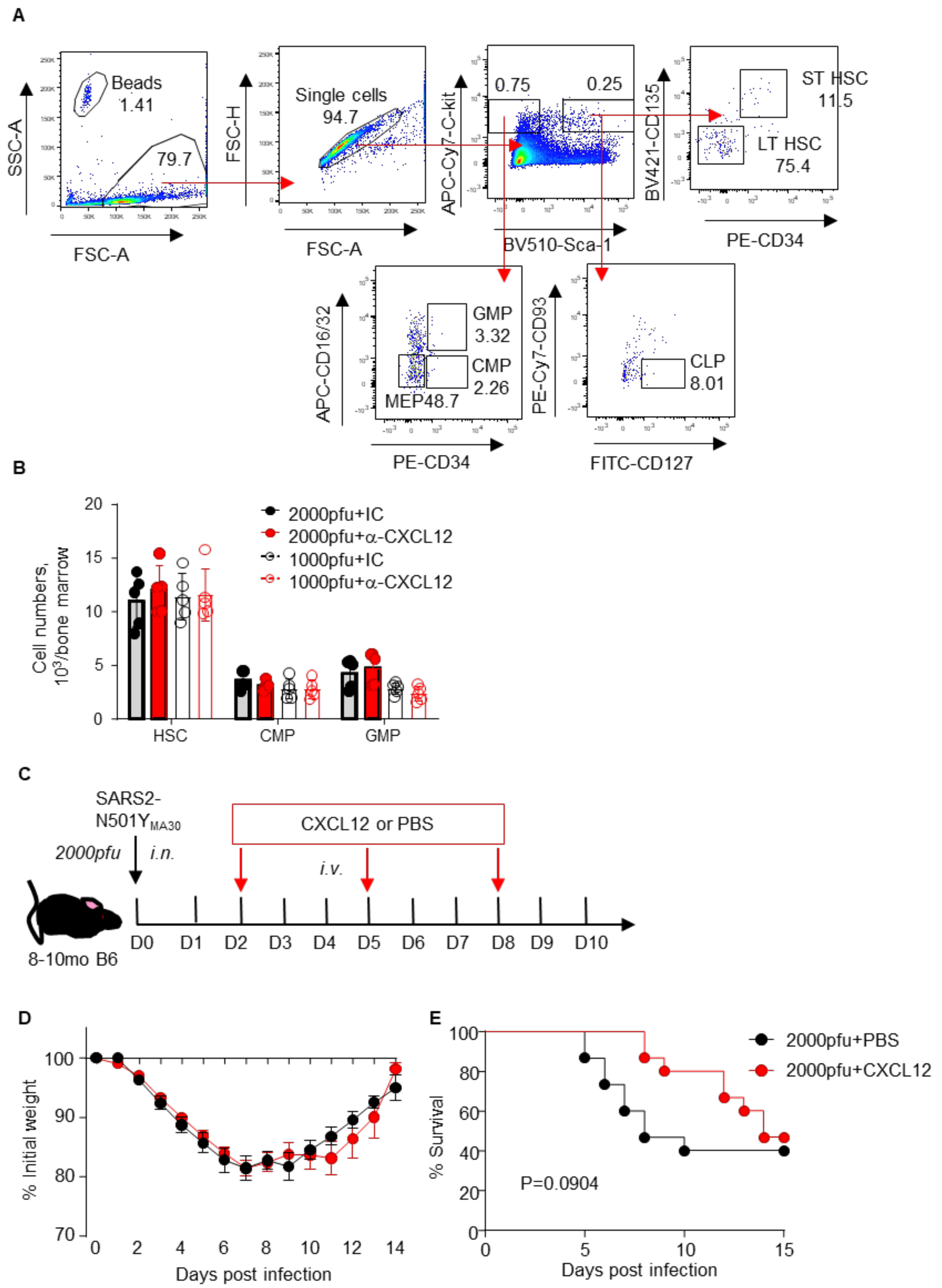

Supplemental Figure 6 CXCL12 blockade does not modify composition of neutrophil precursors in bone marrow. (**A**) Gating strategy for precursors of neutrophils (short-term and long-term hematopoietic stem cells (ST and LT HSC), lympho-myeloid precursors (LMP), granulocyte-monocyte progenitor (GMP)). (**B**) The number of neutrophil precursors in the bone

marrow of 8-10-month-old mice infected with SARS2-N501Y_{MA30} (2000 pfu) was determined by flow cytometry after anti-CXCL12 antibody treatment. n=5. Data are mean±SEM and are representative of two independent experiments. (**C-E**) Middle-aged (8-10-month-old) C57BL/6N mice were infected with 2000 pfu SARS2-N501Y_{MA30} and treated with recombinant mouse CXCL12 or PBS. Weights (**D**) and survival (**E**) are shown (n=15). Data are summary of three independent experiments. Data in **(D)** are mean±SEM.

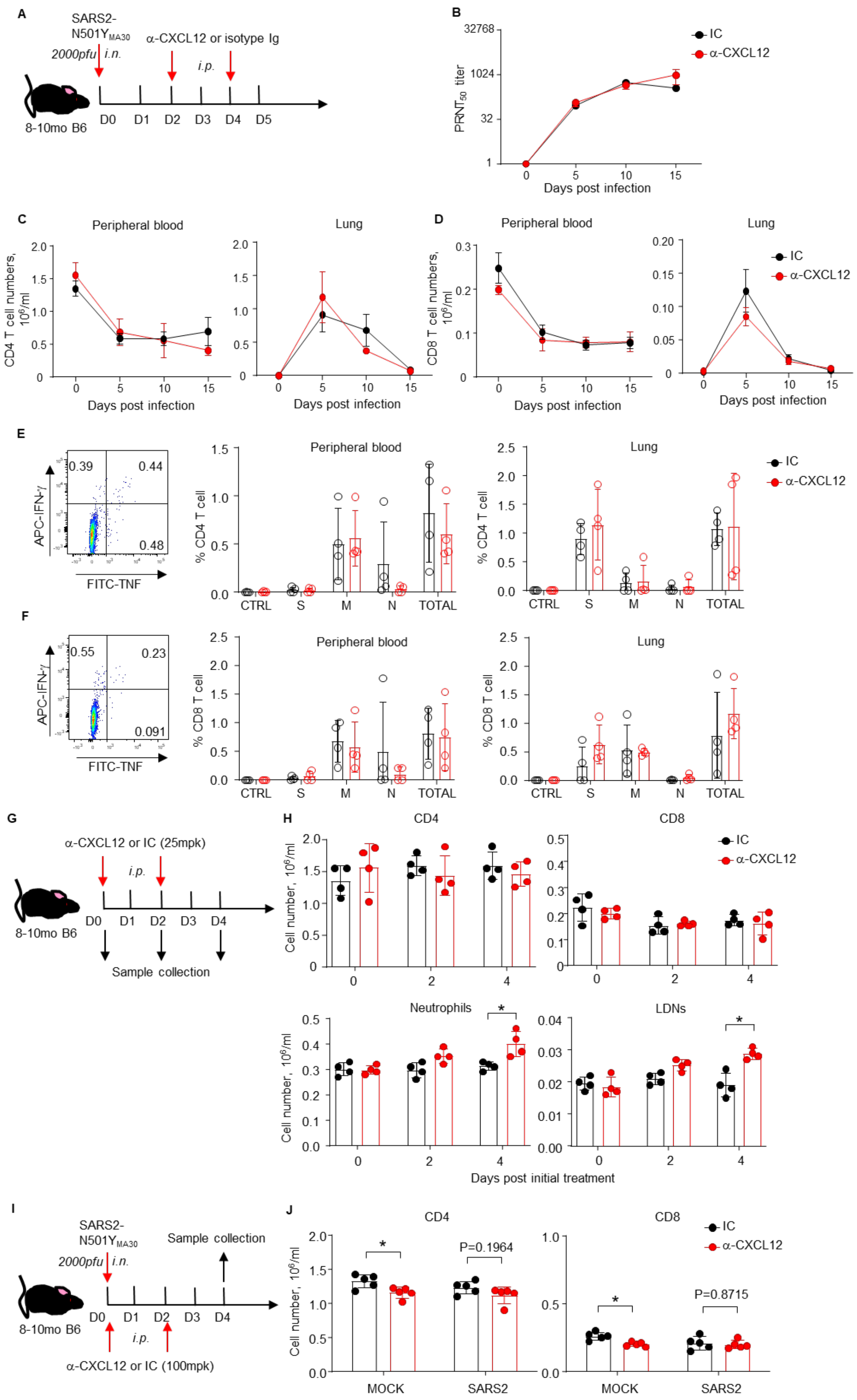
A
SARS2-N501Y
2000pfu i.n.
α-CXCL12 or isotype Ig
i.p.
8-10mo B6
D0 D1 D2 D3 D4 D5
B
PRNT50 titer
IC
α-CXCL12
Days post infection
C
Peripheral blood
Lung
CD4 T cell numbers, 10^6/ml
Days post infection
D
CD8 T cell numbers, 10^6/ml
E
APC-IFN-γ
FITC-TNF
0.39
0.44
0.48
% CD4 T cell
CTRL S M N TOTAL
F
0.55
0.23
0.091
% CD8 T cell
G
α-CXCL12 or IC (25mpk)
Sample collection
H
CD4
CD8
Cell number, 10^6/ml
Neutrophils
LDNs
Days post initial treatment
I
Sample collection
α-CXCL12 or IC (100mpk)
J
P=0.1964
P=0.8715
MOCK
SARS2

57

Supplemental Figure 7. CXCL12 blockade does not affect memory immune responses in SARS2-N501Y$_{MA30}$-infected mice. (**A**) 8-10-month-old C57BL/6N mice were infected with 2000 pfu SARS2-N501Y$_{MA30}$ and treated with anti-CXCL12 or isotype control (IC, isotype Ig) antibody(25mg/kg). (**B**) Titers of serum neutralizing antibody were determined by $PRNT_{50}$ assay at indicated time points. n=5. Data are mean±SEM and are representative of two independent experiments. (**C** and **D**) Numbers of blood and lung CD4 (**C**) and CD8 (**D**) T cells were determined by flow cytometry at the indicated time points. n=5. Data are mean±SEM and are representative of two independent experiments. (**E** and **F**) The frequency of blood and lung memory CD4 (**E**) and CD8 (**F**) T cells (IFN-γ^+TNF$^+$) were determined by in vitro stimulation with SARS-CoV-2 peptide pools (spike protein, S; membrane protein, M; nucleoprotein, N) and flow cytometry at day 30 post infection. n=5. Flow panels are shown for M protein-specific CD4 and CD8 T cell responses obtained from isotype Ig-treated mice. Data are mean±SEM and are representative of two independent experiments. (**G** and **H**) Numbers of blood CD4 and CD8 T cells, neutrophils and LDNs in 8-10-month-old naïve C57BL/6N mice treated with anti-CXCL12 antibody or its isotype control (IC, isotype Ig). *P<0.05. n=4. Data are mean±SEM and are representative of two independent experiments. (**I** and **J**) 8-10-month-old C57BL/6N mice were infected with 2000 pfu SARS2-N501Y$_{MA30}$ or PBS, followed by anti-CXCL12 or isotype control (IC, isotype Ig) antibody treatment, 100mg/kg, at day 0 and 2 post infection. Peripheral blood T cells were determined at day 4 post infection. *P<0.05. n=5. Data are mean±SEM and are representative of two independent experiments. mpk, mg/kg weight.

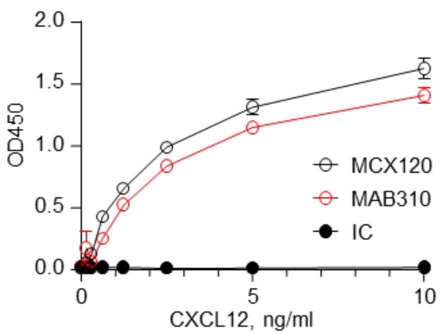

Supplemental Figure 8: Antigen-specificity of anti-CXCL12 antibody. 96-well plates were pre-coated with anti-CXCL12 antibody (MCX120 used for ELISA or MAB310 used for blocking assays, both from R&D) or isotype control antibody (IC). The standard samples included in CXCL12 ELISA kit were then serially diluted and added into pre-coated plates in triplicate. A standard curve was determined following the manufacturer’s instructions.
